# Capacity Analysis of Hybrid Satellite–Terrestrial Systems with Selection Relaying

**DOI:** 10.3390/e26050419

**Published:** 2024-05-13

**Authors:** Predrag Ivaniš, Jovan Milojković, Vesna Blagojević, Srđan Brkić

**Affiliations:** School of Electrical Engineering, University of Belgrade, Bulevar kralja Aleksandra 73, 11000 Belgrade, Serbia; mj205018p@student.etf.bg.ac.rs (J.M.); vesna.golubovic@etf.rs (V.B.); srdjan.brkic@etf.rs (S.B.)

**Keywords:** cooperative relaying, ergodic capacity, low earth orbit (LEO), outage capacity, outage probability, satellite–terrestrial system, shadowed Rice fading

## Abstract

A hybrid satellite–terrestrial relay network is a simple and flexible solution that can be used to improve the performance of land mobile satellite systems, where the communication links between satellite and mobile terrestrial users can be unstable due to the multipath effect, obstacles, as well as the additional atmospheric losses. Motivated by these facts, in this paper, we analyze a system where the satellite–terrestrial links undergo shadowed Rice fading, and, following this, terrestrial relay applies the selection relaying protocol and forwards the information to the final destination using the communication link subjected to Nakagami-*m* fading. For the considered relaying protocol, we derive the exact closed-form expressions for the outage probability, outage capacity, and ergodic capacity, presented in polynomial–exponential form for the integer-valued fading parameters. The presented numerical results illustrate the usefulness of the selection relaying for various propagation scenarios and system geometry parameters. The obtained analytical results are corroborated by an independent simulation method, based on the originally developed fading simulator.

## 1. Introduction

The design of new-generation wireless communication networks which are able to support a wide range of applications and services represents a major challenge. The demands that contemporary wireless networks should answer are enabling a high data rate, providing high reliability, a high security level, both low latency and energy consumption, as well as a high density of user terminals [1].

Contemporary terrestrial wireless networks (5G and beyond) are designed to achieve all of these requirements. However, the quality of the service provided by the terrestrial networks can be reduced due to the limited network coverage and cell overloading, caused by an excessive number of devices being connected to one base station.

Satellite networks as well as aerial ones, jointly referred to as non-terrestrial networks, are foreseen to contribute to coverage extension, multicast capabilities, and increased interoperability with terrestrial networks due to their unique features [2]. The contribution of the satellite segment, achieved through integration with the terrestrial one, is multifold. They are essential for improving 5G network functionality through the offloading of the terrestrial network, achieved by broadcasting content to the edge of the network, or directly to users, as reported in [3]. Furthermore, the role of satellite networks is extremely important in enhancing mobile broadband, as highlighted in [4].

Lately, satellite mega-constellations are becoming an appealing solution for providing global internet coverage and the aforementioned improvement of terrestrial networks. While the higher altitude of the satellite results in a smaller number of satellites needed for global coverage, it also leads to a larger path loss and latency, which represent an issue for many delay-sensitive services. Low earth orbit (LEO) satellite systems represent appealing solutions, although the maintenance of LEO systems is challenging as satellites orbit with a very high velocity at lower heights, while the diameter of a single beam is small. The stationary end user, located at Earth’s surface, lies within a single beam for a short period of time [5,6], which results in frequent handover procedures and a constant need for the adaptation of elevation angles [7].

An initial estimation of the total system throughput for three mega-constellation satellite systems (OneWeb, Telesat, and SpaceX) was provided in [5]. The coverage and capacity analysis of the LEO network that supports the Internet of Things was presented in [8]. In [9], it was shown how the optimization of the network parameters can increase the capacity of LEO satellite systems. The authors of paper [10] minimized the number of satellites in the LEO constellation by using the optimization of the total backhaul capacity of user terminals in the LEO network. The estimation of the total capacity of the Starlink satellite system was provided in [11], and the analysis was given for the typical system parameters and typical propagation conditions.

In addition to the system resources allocation, which dominantly affects the system capacity, accurate channel modeling is of the highest importance, as it is a necessary input to any resource optimization and performance verification strategy. An adequate statistical model of the satellite–terrestrial channel is important for correct capacity analysis, as atmospheric conditions result in the stochastic attenuation of the signal [5]. For the case of land mobile systems, a user experiences multipath and shadowing effects, and the shadowed Rice model was widely accepted to describe the statistics of the channel between the satellite and terrestrial user [12].

It is well known that relay employment can significantly increase the capacity and improve the link reliability of wireless communications systems [13,14,15,16]. Similar approaches were proposed for the satellite–terrestrial systems, and corresponding performances were provided in [17,18,19,20,21,22,23,24,25,26,27,28,29,30]. An overview of these papers is given in Section 1.1.

In this paper, we consider the scenario where satellite–terrestrial links are under the impact of shadowed Rice fading, and terrestrial relays forward the information broadcasted by the satellite to the final destination using the communication link subjected to Nakagami-*m* fading. Our main contributions are the exact closed-form expressions for the outage probability, outage capacity, and ergodic capacity, obtained in polynomial–exponential form for the integer-valued fading parameters. The numerical results are presented for various propagation scenarios and system geometry parameters. The obtained results are verified through the use of the independent Monte Carlo simulation method, based on the originally developed simulator.

### 1.1. Related Work

It is a well-established fact that the performance of terrestrial mobile networks can be improved using cooperative relaying techniques [13]. In the fundamental paper [14], it was shown that the outage probability, which measures the robustness of the transmissions when the fading is present in the wireless channel, can be reduced if the appropriate cooperation protocol is applied. In various wireless networks, the relays are applied to forward the received signal from a source to an intended destination, and amplify-and-forward (AF) and decode-and-forward (DF) relaying protocols are widely used relaying protocols [15]. Furthermore, it has been shown that the system performance can be additionally improved if the cooperation strategy is adaptive to the instantaneous channel conditions. If the feedback from the destination is not available, the selection relaying strategy adapts the transmitted signal at the relay based on the measurements of the corresponding signal–noise ratio (SNR) [14,16].

In land mobile satellite systems, the communication links can be unstable due to the existence of obstacles in the line of sight (LOS) between the satellite and terrestrial user, losses related to low elevation angles, and/or additional rain- or fog-induced atmospheric losses. Therefore, a masking effect can appear as a result of the obstacles and shadowing. As a result, the LOS signal component can be attenuated, therefore degrading the performance of the satellite–terrestrial link. This effect can be compensated by using terrestrial relays, leading to the concept of a hybrid satellite–terrestrial relay network (HSTRN) being proposed in [17]. The performance analysis of the HSTRN with an AF relaying protocol and maximal ratio combining (MRC) being applied at the destination was given in [18,19]. In [20], the ergodic capacity and outage probability expressions were derived for the HSTRN with AF protocol. The performance of adaptive transmission in the HSTRN with DF relaying was analyzed in [21]. The outage probability and throughput of a system with AF and multiple relays were determined in [22]. In these papers, it was assumed that the satellite–terrestrial link undergoes shadowed Rice fading, while the terrestrial link typically undergoes Rayleigh fading. The upper bound for ergodic capacity and the approximation of the outage probability were derived in [23] for the case where the communication between the satellite and terrestrial user is improved via the use of reconfigurable intelligent surfaces.

The performance analysis of the multiuser case of the HSTRN with fixed AF relaying was given in [24]. The ergodic capacity of the cognitive HSTRN was determined in [25] for the case where there is no direct link between the geostationary satellite and the destination. The outage analysis of the system with multiple terrestrial relays that apply threshold-based DF transmission protocols was given in [26] for the case without the direct link between the satellite and terrestrial user. In [27], it was assumed that the aerial relays (high- altitude platforms and unmanned aerial vehicles) assist satellite–terrestrial communication. The closed-form outage probability expressions were presented in the paper [28] for the case when two remote terrestrial users mutually communicate via a LEO mega-constellation satellite system, where terrestrial, aerial, or satellite relays forward the information broadcasted by the satellite to the other satellite. In the papers [24,25,26,27,28], the analytical results were obtained via the use of the assumption that the link between the satellite and the terrestrial user is modeled with shadowed Rice fading, that the terrestrial link is modeled by Nakagami-*m* fading, and that the fading parameters in both the satellite and terrestrial link are integer valued. In the more recent papers [29,30,31,32], the performance analysis of the non-orthogonal multiple access (NOMA)-based satellite–terrestrial-integrated network was given, based on a channel model that incorporates the antenna array pattern and attenuation caused by rain, resulting in higher accuracy.

In [33], the outage probability analysis was presented for two cooperation strategies, where there is a direct link between the satellite and terrestrial user, and the communication is assisted with the terrestrial relay. However, the outage probability expressions were not presented in the closed form, and the system geometry was not considered. Also, the presented simulated method was based on a temporally uncorrelated time series. For the same system configuration and the case of integer-valued fading parameters, in our recent conference paper [34], we derived a closed-form expression for the outage probability. In another conference paper [35], we developed a simulator of shadowed Rice fading that generates the corresponding fading samples with arbitrary temporal properties.

In this paper, we determine the capacity of the LEO link assisted with the selection relaying protocol, which adapts the transmitted signal depending on the SNR at the relay. Our work is motivated by the increasing need for the efficient usage of available spectral resources due to the growing number of satellite communication links, as well as the inherent characteristics of satellite links that can be blocked due to heavy shadowing effects. The selection relaying protocol investigated in this paper is a possible method for making satellite networks more efficient, as it enables communication in severely impaired propagation conditions, subsequently increasing the capacity of the entire network. It represents a low complexity solution, provides superior performance when compared to fixed relaying, and does not require feedback information from the end user like incremental relaying does [14]. It should be noted that our work is primarily aimed at investigating the experience improvement of satellite users, and it does not take into account what effect communication overhead, created by the communication link between the relay and the user, has on the HSTRN. Although we do not claim that selection relaying is the optimal way to combine terrestrial and satellite infrastructures, it provides considerable communication advantages if the satellite users are prioritized.

### 1.2. Contribution and Organization

In this paper, we provide a performance analysis of a satellite–terrestrial system, where there is a direct link between the satellite and terrestrial user, and the communication is supported by a relay that adapts the transmission signal based on the selection relaying protocol. Our main goal is to identify scenarios where terrestrial relays can increase the capacity of the system. The contributions of this paper are as follows:

- We propose a model of the satellite–terrestrial communication system, where the transmitted signal at the relay depends on the result of the comparison of the received SNR with the arbitrary local threshold;
- In this paper, we derive the novel analytical expressions for the outage probability for the arbitrary threshold at the relay, which can be different from the threshold at the destination, extending our previous results published in [34], which were based on more general probability density functions. Additionally, we derive novel expressions for the outage capacity and ergodic capacity, providing the corresponding numerical results;
- We derive the analytical expressions for the probability density function of the received SNR at the destination for the given threshold at the relay;
- We derive the analytical expressions for the outage capacity of the system, which is a relevant performance measure for applications with delay constraints;
- We derive the relevant analytical expressions for the ergodic capacity of the analyzed system for applications with no delay requirements;
- All analytical expressions derived in the paper are given in polynomial–exponential form, and they are valid for the general case of the shadowed Rice fading environment with the integer-valued fading parameter at the satellite–terrestrial links and Nakagami-*m* fading environment at the terrestrial link;
- The derived expressions are general and applicable to a transmission system with arbitrary system parameters;
- We propose a novel method for generating the time series that corresponds to the time-varying channel gains in satellite–terrestrial links as an improved version of the simulation method that we proposed in [35]. In this paper, we use an improved simulation method that includes the terrestrial component, deriving the corresponding temporal autocorrelation function of the complex channel gain in the satellite–terrestrial link;
- Analytical results are confirmed using an independent Monte Carlo simulation method, and the corresponding numerical results are presented for the various propagation scenarios and typical parameters of the active LEO satellite systems.

The analytical results for the outage capacity and ergodic capacity are derived in the closed form, and, to the best of our knowledge, these expressions are not presented in the available literature. The significance of the closed-form expressions lies in the fact that they are easy to compute and can be used directly as input arguments for network resource optimizations, contrary to the results of computationally hungry simulations. For example, based on the outage probability values, the network optimizer can find a suitable relay location that maximizes the user’s experience. Similarly, achievable communication capacities can be used for optimizing satellite power allocation and handover execution strategies, as discussed in [36]. The aforementioned applications of our results are out of the scope of this paper, and they are intended for future extensions of this work.

In the following sections, we present a complete analysis and the applied simulation method in detail. In Section 2, the system and channel models are presented, and the list of symbols is shown in Table 1. The outage probability and the outage capacity of the analyzed system are described in Section 3. The ergodic capacity of the system with a cooperative relay is analyzed in Section 4. The numerical results and discussion are provided in Section 5, where the derived expressions are confirmed through the use of an independent simulation method. Section 6 concludes the paper.

## 2. System and Channel Model

**Figure 1:**
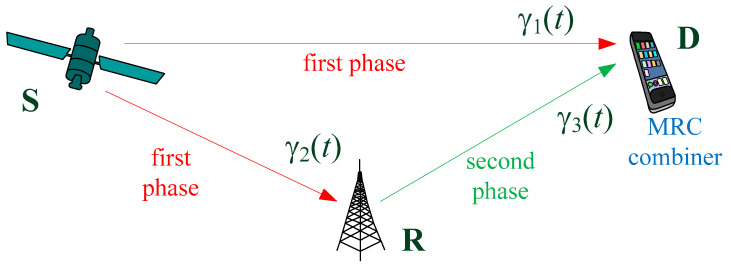
MRC combiner and time slots.

The hybrid satellite–terrestrial relay network is shown in Figure 1. The satellite S sends the same signal to the terrestrial users—the mobile destination D and the relay R. The relay R uses a decode-and-forward technique with selection relaying, i.e., it retransmits the received signal from the satellite S to the destination D, with the additional adaptation to the instantaneous channel conditions. Furthermore, we assume that the multi-beam satellite is placed in the LEO orbit at a certain altitude *H*, and the diameter of the beam, denoted by *L*, is much smaller than the satellite altitude.

The following strategy of cooperative relaying is applied:

- In the first half of the signalization interval, the source sends a signal to the receivers at the destination and at the relay. The instantaneous received SNR in the S-D link in an arbitrary time instant *t* is denoted by γ_1_(*t*), and the instantaneous received SNR in the S-R link is denoted by γ_2_(*t*).
- After the first half of the signalization interval, the relay performs decoding. If the decoding is successful, in the second half of the signalization interval, the receiver at the destination receives the signal from the relay, with the instantaneously received SNR in the R-D link being denoted by γ_3_(*t*). Otherwise, the relay is silent (it does not send any signal). The decoding at the relay is usually considered successful in the time instant *t* if the received SNR is larger than the predefined threshold, denoted by γ_th,R_ [14]. The value of the threshold depends on the applied modulation and coding scheme at the corresponding communication link, as well as the receiver sensitivity.
- The signals received in the first and the second half of the signalization interval are combined. In this paper, we assume that MRC is applied at the destination receiver. Therefore, the SNR at the output of the MRC combiner is obtained as the sum of the SNRs in two intervals [33] as follows:

(1) $$\gamma (t)=\left\{\begin{array}{cc} \gamma_{1}(t), & \gamma_{2}(t)<\gamma_{\mathrm{th},R},\\ \gamma_{1}(t)+\gamma_{3}(t), & \gamma_{2}(t)\geq \gamma_{\mathrm{th},R}. \end{array}\right.$$

The instantaneous SNR at the *i*-th receiver of the satellite–ground station link can be obtained as [19,20,21,28]

(2) $$\gamma_{i}(t)={\bar{\gamma }}_{i}{\left|h_{i}(t)\right|}^{2},$$

where ${\bar{\gamma }}_{i}$ denotes the corresponding average SNR, and *h_i_*(*t*) denotes the time-varying complex channel gain in the *i*-th communication channel. As defined before, the channel gain between the satellite and the destination (mobile user) is the first one (*i* = 1), the channel gain between the satellite and the relay is the second one (*i* = 2), and the channel gain between the relay and the destination is the third one (*i* = 3).

**Figure 2:**
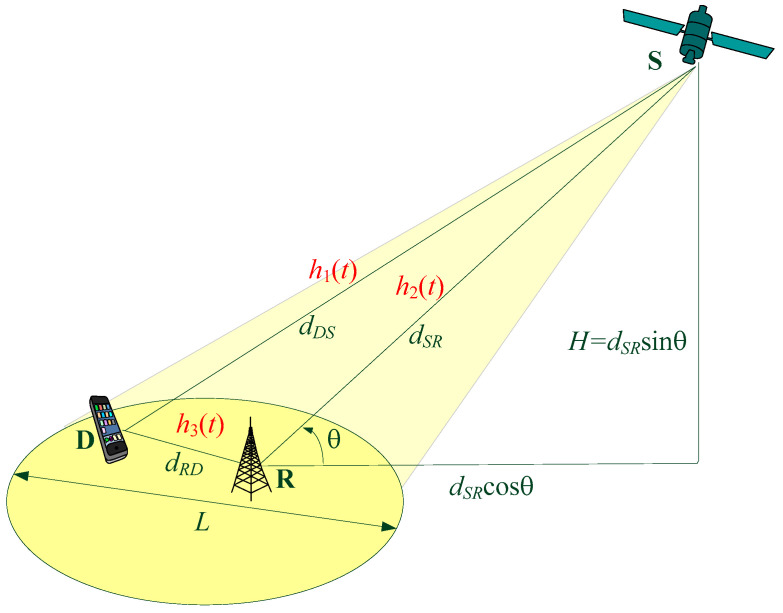
The system geometry.

The average SNR depends on the system parameters, namely the transmit power, the average power of the noise, the distance over the link between the transmitter and the receiver, and the corresponding path loss factor. Taking into account the system geometry presented in Figure 2, we can determine the average SNRs as follows [28]:

(3) $${\bar{\gamma }}_{1}=\frac{P_{S}}{\sigma^{2}d_{SD}^{n_{SD}}},\;{\bar{\gamma }}_{2}=\frac{P_{S}}{\sigma^{2}d_{SR}^{n_{SR}}},\;{\bar{\gamma }}_{3}=\frac{P_{R}}{\sigma^{2}d_{RD}^{n_{RD}}}.$$

In the above equations, the average transmitted power at the satellite is denoted by *P_S_*, the average transmitted power at the relay is denoted by *P_R_*, *d*_SD_ denotes the distance between the satellite and the destination, *d*_SR_ denotes the distance between the satellite and the relay, and *d*_RD_ denotes the distance between the relay and the destination. The corresponding path loss factors are denoted by *n*_SD_, *n*_SR_, and *n*_RD_. We assume that the noise power values at the destination and at the relay are equal and are denoted by σ^2^.

Due to the relatively small beam diameter (*L* ≪ *H*), it is reasonable to assume that the elevation angles at the destination and relay satisfy the condition θ_R_ = θ_D_ = θ, and the corresponding distances are approximately the same, i.e., *d_SD_* ≈ *d_SR_* = *H*/sin(θ).

*A.*  *Satellite–terrestrial channel*

The temporal properties of the instantaneous SNR in the *i*-th channel are completely determined with the time-varying complex channel gain *h_i_*(*t*). In the available literature, various channel models were developed to describe the time-varying complex channel gain in a narrowband land mobile satellite channel. It is usually considered that the fluctuations are the result of many weak scattered components (multipath fading) and the random variations of the total power of the multipath components (shadowing) [37].

Available channel models are usually developed to fit the first- and second-order statistics of the physical channels, with the probability density function (PDF) and the autocorrelation function (ACF). In Loo’s model, the amplitude of the LOS component is modeled via the use of the random variable with log-normal distribution [38]. Although this model corresponds to the measurement results available in the literature, the complicated expressions for the first- and second-order statistics limit its application in mathematical analysis. Furthermore, it was shown that the shadowed Rice model [12,39] is a simpler but very accurate channel model for narrowband land mobile satellite channels. In this model, it is assumed that the channel gain is determined as a summation of the gamma-shadowed LOS component and scattered components.

In this paper, we will use the shadowed Rice model, where the time-varying complex gain of the satellite–terrestrial channels will be modeled as [12]

(4) $$h_{i}(t)=a_{i}(t)e^{-j\alpha_{i}(t)}+z_{i}(t)e^{{j\varsigma }_{0,i}},\;i=1,2.$$

In the above expression, the first term corresponds to the time-varying scattering component, and the second term corresponds to the LOS component. The phase α*_i_*(*t*) of the scattering component is uniformly distributed, while the corresponding amplitude *a_i_*(*t*) exhibits Rayleigh distribution as follows:

(5) $$f_{A}(a_{i})=\frac{a_{i}}{b_{0,i}}e^{-\frac{a_{i}^{2}}{2b_{0,2}}},a_{i}\geq 0,\;i=1,2,$$

where 2*b*_0,*i*_ denotes the average power of the scattering component.

The second term in Equation (4) corresponds to the LOS component, with the deterministic phase ζ_0,*i*_ and Nakagami-*m* distributed envelope *z_i_*(*t*) as follows:

(6) $$f_{Z}\left(z_{i}\right)=\frac{{{2m}_{i}}^{m_{i}}}{\Gamma (m_{i})\Omega_{i}^{m_{i}}}{z_{i}}^{2m_{i}-1}\exp\left(-\frac{m_{i}}{\Omega_{i}}{z_{i}}^{2}\right),\;i=1,2,$$

where Ω*_i_*denotes the average power of the LOS component in *i*-th channel and *m_i_* denotes the corresponding Nakagami fading parameter.

The corresponding probability density function of the normalized power gain in the *i*-th satellite–terrestrial channel, denoted by λ*_i_*(*t*) = |*h_i_*(*t*)|^2^, can be provided using the following [12]:

(7) $$f_{\Lambda_{i}}(\lambda_{i})={\left(\frac{2b_{0,i}m_{i}}{2b_{0,i}m_{i}+\Omega_{i}}\right)}^{m}\frac{1}{2b_{0,i}}e^{-\frac{\lambda_{i}}{2b_{0,i}}}{\;}_{1}F_{1}\left(m_{i};1;\frac{\Omega_{i}\lambda_{i}}{2b_{0,i}(2b_{0,i}m_{i}+\Omega_{i})}\right),\lambda_{i}\geq 0,\;i=1,\;2,$$

where _1_F_1_( ; · ; ) denotes the confluent hypergeometric function of the first kind.

In the special case of integer-valued parameters *m*_1_ and *m*_2_, the hypergeometric function can be represented in the form of the finite summation of polynomial–exponential terms [28], resulting in a simplified PDF expression as follows:

(8) $$f_{\Lambda_{i}}(\lambda_{i})=\alpha_{i}\sum_{k=0}^{m_{i}-1}\varsigma_{i}(k)\lambda_{i}^{k}e^{-\left(\beta_{i}-\delta_{i}\right)\lambda_{i}},\;i=1,\;2,$$

where $\varsigma_{i}(k)={\left(-1\right)}^{k}{\left(1-m_{i}\right)}_{k}\delta_{i}^{k}/{\left(k!\right)}^{2},$ and (*t*)*_k_*= *t*(*t* + 1)*···*(*t* + *k −* 1) denotes the Pochhammer symbol, and the following substitutions are to be applied:

(9) $$\alpha_{i}=\frac{1}{2b_{0,i}}{\left(\frac{2b_{0,i}m_{i}}{2b_{0,i}m+\Omega_{i}}\right)}^{m_{i}},\;\beta_{i}=\frac{1}{2b_{0,i}},\;\delta_{i}=\frac{\Omega_{i}}{2b_{0,i}(2b_{0,i}m_{i}+\Omega_{i})}.\;$$

Finally, by using the linear transformation of random variables $\gamma_{i}(t)={\bar{\gamma }}_{i}\times \lambda_{i}$, we obtain the PDFs of the instantaneous SNRs, applicable for the S-D and S-R links, as follows:

(10) $${f_{\Gamma }}_{i}(\gamma_{i})=\alpha_{i}\sum_{k=0}^{m-1}\frac{\varsigma_{i}(k)}{{\bar{\gamma }}_{i}^{k+1}}\gamma_{i}^{k}e^{-\frac{\beta_{i}-\delta_{i}}{{\bar{\gamma }}_{i}}\gamma_{i}},\;i=1,2.$$

The second-order statistics of the shadowed Rice channel are dominantly determined using the scattering component, as empirical observations have shown that the LOS varies slowly. Therefore, the temporal properties of the channel gain *h_i_*(*t*) (*i* = 1, 2) are dominantly determined using the ACF of the scattering component.

Furthermore, in the case of isotropic scattering [40], the time-varying properties of the scattering component with the Rayleigh distribution and the average power 2*b*_0,*i*_ can be successfully described using the autocorrelation function as follows:

(11) $$R_{a,i}(\tau )=2b_{0,i}J_{0}(2\pi f_{Dm}\tau ),$$

where *f_D_*_m_ denotes the maximum Doppler frequency. The above expression was given in the book [41], where the second-order statistics for the isotropic scattering are presented in detail. The second-order statistics for the diversity systems were described in [42,43] for the case of Rayleigh distribution, and in [44] for the case of Nakagami-*m* distribution (both distributions will be used in our simulation model).

*B.*  *Channel model in terrestrial links*

In this paper, we assume that the envelope of the time-varying channel gain between R and D follows Nakagami distribution. Therefore, the first-order statistics of the normalized power gain in the R-D terrestrial channel λ_3_(*t*) = |*h*_3_(*t*)| can be modeled using a random variable with a Gamma distribution as follows:

(12) $${f_{\Lambda }}_{3}(\lambda_{3})=\frac{m_{3}^{m_{3}}\lambda_{3}^{m_{3}-1}}{\Omega^{m_{3}}\Gamma (m_{3})}e^{-\frac{m_{3}\lambda_{3}}{\Omega_{3}}},$$

where Ω_3_E{λ_3_} denotes is the average power gain, *m*_3_ denotes the corresponding fading parameter, and Γ(·) denotes the Gamma function.

The Nakagami-*m* fading model is more suitable for mathematical manipulations when compared to the widely accepted Rice fading model. If the Rice parameters (the ratio between the power of LOS and the power of the scattered components, denoted by *K*, and the power of the scattered waves, denoted by σ_S_^2^) are of a certain value, then the Nakagami-*m* parameters *m*_3_ and $\Omega_{3}$ can be obtained as $m_{3}=(1+K^{2})/(2K+1)$ and ${\sigma_{S}}^{2}=\Omega_{3}\left(1-\sqrt{1-1/m_{3}}\right)/2$ [40].

By using the linear transformation of random variables $\gamma_{i}(t)={\bar{\gamma }}_{i}\times \lambda_{i}$ and the equations given in the previous section, it is easy to obtain the PDFs of the instantaneous SNRs applicable for the R-D link as follows:

(13) $$f_{\Gamma_{3}}(\gamma_{3})=\frac{m_{3}^{m_{3}}\gamma_{3}^{m_{3}-1}}{\Omega_{3}^{m_{3}}{\bar{\gamma }}^{m_{3}}\Gamma (m_{3})}e^{-\frac{m_{3}\gamma_{3}}{\Omega_{3}{\bar{\gamma }}_{3}}}.$$

The ACF of the Nakagami-*m* fading envelope was given with the expression [45]

(14) Rh(τ)=ΩΓ2(m3+1/2)m3Γ2(m3)F12−12,−12;m3;J02(2πfDsτ),

where _2_F_1_(·, ·; ·; ·) denotes the hypergeometric function [46] and *J*_0_(.) denotes the Bessel function of the first kind and the zeroth order. In [47], the authors argued that the rank statistics of the Nakagami-*m* fading envelope are approximately the same for various values of the fading parameter *m*_3_. As the rank autocorrelation coefficient is known for Rayleigh fading (*m*_3_ = 1), we will assume that the same expression is valid for higher values of *m*_3_, thus determining the second-order statistics in the terrestrial link.

## 3. Outage Capacity

An important feature describing wireless systems with slow fading is the outage capacity, which is defined as the maximum data rate that can be achieved in the channel with the outage probability *P_out_*(γ_th_).

In the analyzed system, both satellite–terrestrial links (S-D and S-R) are active only in the first half of the signalization interval, while the terrestrial link R-D is active only in the second half of the signalization interval. This fact effectively doubles the signaling rate when compared with the system without relaying, where the communication link S-D is used during the whole signalization interval. The efficiency of the use of the available bandwidth is reduced twice in the system with relaying, and the instantaneous capacity of the proposed system is defined as [48]

(15) $$C(t)=\left\{\begin{array}{cc} 0.5\log_{2}\left(1+\gamma_{1}(t)\right), & \gamma_{2}(t)<\gamma_{\mathrm{th},R},\\ 0.5\log_{2}\left(1+\gamma_{1}(t)+\gamma_{3}(t)\right), & \gamma_{2}(t)\geq \gamma_{\mathrm{th},R}. \end{array}\right.$$

The outage capacity could be estimated through separately deriving the outage probability for the S-D and R-D channels for the given value γ_th,R_. However, such an analysis could not provide complete information about the impact of the S-R link. By using the expression for the instantaneous SNR at the output of the MRC combiner, denoted by γ(*t*), the above expression can be rewritten in a more compact form as follows:

(16) $$C(t)=0.5\log_{2}\left(1+\gamma (t)\right),$$

and the outage capacity can be expressed by equation [49].

(17) $$C_{out}(\gamma_{th}\left|\gamma_{th,R}\right.)=0.5(1-P_{out}(\gamma_{th}\left|\gamma_{th,R}\right.))\log_{2}(1+\gamma_{th})$$

The outage probability can be obtained using the following identity [33]:

(18) $$P_{out}(\gamma_{th}\left|\gamma_{th,R}\right.)=\int_{0}^{\gamma_{th,R}}f_{\Gamma_{2}}(\gamma_{2})d\gamma_{2}\int_{0}^{\gamma_{th}}f_{\Gamma_{1}}(\gamma_{1})d\gamma_{1}+\int_{\gamma_{th,R}}^{\infty }f_{\Gamma_{2}}(\gamma_{2})d\gamma_{2}\int_{0}^{\gamma_{th}}f_{\Gamma_{1}}(\gamma_{1})\int_{0}^{\gamma_{th}-\gamma_{1}}f_{\Gamma_{3}}(\gamma_{3})d\gamma_{3}\;d\gamma_{1}.$$

The second integral in the above expression can be expressed using the cumulative distribution function (CDF) of the SNR is the S-D link, and by using Equation (10), we obtain that

(19) $$I_{1}=\int_{0}^{\gamma_{th}}f_{\Gamma_{1}}(\gamma_{1})d\gamma_{1}=1-\int_{\gamma_{th}}^{\infty }f_{\Gamma_{1}}(\gamma_{1})d\gamma_{1}=1-\alpha_{1}\sum_{k=0}^{m_{1}-1}\frac{\varsigma_{1}(k)}{{\bar{\gamma }}_{1}^{k+1}}\int_{\gamma_{th}}^{\infty }\gamma_{1}^{k}e^{-\frac{\beta_{1}-\delta_{1}}{{\bar{\gamma }}_{1}}\gamma_{1}}d\gamma_{1}.$$

After applying the definition for the upper incomplete gamma function [46]

(20) $$\Gamma \left(s,x\right)=\int_{x}^{\infty }t^{s-1}e^{-t}dt,$$

we can write

(21) $$I_{1}=1-\alpha_{1}\sum_{k_{1}=0}^{m_{1}-1}\frac{\varsigma_{1}(k_{1})}{{\bar{\gamma }}_{1}^{k_{1}+1}}{\left(\frac{\beta_{1}-\delta_{1}}{{\bar{\gamma }}_{1}}\right)}^{-(k_{1}+1)}\Gamma \left(k_{1}+1,\frac{\beta_{1}-\delta_{1}}{{\bar{\gamma }}_{1}}\gamma_{th}\right),\;i=1,2.$$

Finally, through using the well-known identity [46]

(22) $$\Gamma \left(s,x\right)=\int_{x}^{\infty }t^{s-1}e^{-t}dt=(s-1)!e^{-x}\sum_{p_{1}=0}^{s-1}\frac{x^{p_{1}}}{p_{1}!},$$

we obtain the solution of the integral in the polynomial–exponential form.

(23) $$I_{1}=1-\alpha_{1}e^{-\frac{\beta_{1}-\delta_{1}}{{\bar{\gamma }}_{1}}\gamma_{th}}\sum_{k_{1}=0}^{m_{1}-1}\frac{\varsigma_{1}(k_{1})}{{\bar{\gamma }}_{1}^{k_{1}+1}}\sum_{p_{1}=0}^{k_{1}}\frac{k_{1}!}{p_{1}!}{\left(\frac{\beta_{1}-\delta_{1}}{{\bar{\gamma }}_{1}}\right)}^{-(k_{1}+1-p_{1})}\gamma_{th}^{p_{1}}.$$

Using the same approach, we can solve the first integral in Equation (17), related to the S-R link, as follows:

(24) $$I_{2}=\int_{0}^{\gamma_{th,R}}f_{\Gamma_{2}}(\gamma_{2})d\gamma_{2}=1-\alpha_{2}\sum_{k_{2}=0}^{m_{2}-1}\frac{\varsigma_{2}(k_{2})}{{\bar{\gamma }}_{2}^{k_{2}+1}}\int_{\gamma_{th,R}}^{\infty }\gamma_{2}^{k_{2}}e^{-\frac{\beta_{2}-\delta_{2}}{{\bar{\gamma }}_{2}}\gamma_{2}}d\gamma_{2},$$

which can be also represented in the polynomial–exponential form.

(25) $$I_{2}=1-\alpha_{2}e^{-\frac{\beta_{2}-\delta_{2}}{{\bar{\gamma }}_{2}}\gamma_{\mathrm{th},R}}\sum_{k_{2}=0}^{m_{2}-1}\frac{\varsigma_{2}(k_{2})}{{\bar{\gamma }}_{2}^{k_{2}+1}}\sum_{p_{2}=0}^{k_{2}}\frac{k_{2}!}{p_{2}!}{\left(\frac{\beta_{2}-\delta_{2}}{{\bar{\gamma }}_{2}}\right)}^{-(k_{2}+1-p_{2})}\gamma_{\mathrm{th},R}^{p_{2}}.$$

Also, it is obvious that we can write

(26) $$I_{3}=\int_{\gamma_{th,R}}^{\infty }f_{\Gamma_{2}}(\gamma_{2})d\gamma_{2}=\alpha_{2}e^{-\frac{\beta_{2}-\delta_{2}}{{\bar{\gamma }}_{2}}\gamma_{\mathrm{th},R}}\sum_{k_{2}=0}^{m_{2}-1}\frac{\varsigma_{2}(k_{2})}{{\bar{\gamma }}_{2}^{k_{2}+1}}\sum_{p_{2}=0}^{k_{2}}\frac{k_{2}!}{p_{2}!}{\left(\frac{\beta_{2}-\delta_{2}}{{\bar{\gamma }}_{2}}\right)}^{-(k_{2}+1-p_{2})}\gamma_{\mathrm{th},R}^{p_{2}}.$$

By using the approach presented in the Appendix A, the double integral

(27) $$I_{4}=\int_{0}^{\gamma_{th}}f_{\Gamma_{1}}(\gamma_{1})\int_{0}^{\gamma_{th}-\gamma_{1}}f_{\Gamma_{3}}(\gamma_{3})d\gamma_{3}\;d\gamma_{1}$$

can be represented in the following polynomial–exponential form:

(28) $$\begin{array}{ll} I_{4}=1 & -\alpha_{1}e^{-B\gamma_{th}}\sum_{k=0}^{m_{1}-1}A_{k}\sum_{j=0}^{k}\frac{k!}{j!}\frac{\gamma_{th}^{j}}{B^{k-j+1}}+\alpha_{1}\sum_{k=0}^{m_{1}-1}\sum_{p=0}^{m_{3}-1}\sum_{q=0}^{p}\left(\begin{array}{c} p\\ q \end{array}\right)\frac{{\left(-1\right)}^{q}A_{k}C^{p}}{p!}\gamma_{th}^{p-q}e^{-C\gamma_{th}}\\ & \times \left[\frac{(k+q)!}{{\left(B-C\right)}^{k+q+1}}-e^{-(B-C)\gamma_{th}}\sum_{j=0}^{k+q}\frac{(k+q)!}{j!}\frac{\gamma_{th}^{j}}{{\left(B-C\right)}^{k+q-j+1}}\right], \end{array}$$

where

(29) $$A_{k}=\frac{\varsigma_{1}(k)}{{\bar{\gamma }}_{1}^{k+1}},\;B=\frac{\beta_{1}-\delta_{1}}{{\bar{\gamma }}_{1}},\;C=\frac{m_{3}}{\Omega_{3}{\bar{\gamma }}_{3}}.$$

The outage probability can be derived by combining Equations (21), (25), (26) and (28) as follows:

(30) $$P_{out}(\gamma_{th})=I_{2}\times I_{1}+I_{3}\times I_{4},$$

and, if we define the following constants:

(31) $$K_{i1}(k_{i},p_{i})=\frac{k_{i}!}{p_{i}!}\frac{\alpha_{i}\varsigma_{i}(k){\left(\beta_{i}-\delta_{i}\right)}^{p_{i}-k_{i}-1}}{{\bar{\gamma }}_{i}^{p}},\;K_{i2}=\frac{\beta_{i}-\delta_{i}}{{\bar{\gamma }}_{i}},\;i=1,2,\;K_{41}(k,p)=\frac{k!}{p!}\frac{\;\alpha_{1}A_{k}}{{K_{12}}^{k-p+1}},K_{42}(k,p,q)=\left(\begin{array}{c} p\\ q \end{array}\right)\frac{(k+q)!}{p!}\frac{{\left(-1\right)}^{q}\alpha_{1}A_{k}C^{p}}{{\left(K_{12}-C\right)}^{k+q+1}},\;\;K_{43}(k,p,q,j)=\left(\begin{array}{c} p\\ q \end{array}\right)\frac{(k+q)!}{p!j!}\frac{{\left(-1\right)}^{q}\alpha_{1}A_{k}C^{p}}{{\left(K_{12}-C\right)}^{k+q-j+1}},$$

then the outage probability can be represented as a finite summation of the polynomial–exponential terms.

(32) $$\begin{array}{ll} P_{out} & (\gamma_{th}\left|\gamma_{th,R}\right.)=1-\sum_{k_{1}=0}^{m_{1}-1}\sum_{p_{1}=0}^{k_{1}}K_{11}(k_{1},p_{1})\gamma_{th}^{p_{1}}e^{-K_{12}\gamma_{th}}-\sum_{k_{2}=0}^{m_{2}-1}\sum_{p_{2}=0}^{k_{2}}K_{21}(k_{2},p_{2})\gamma_{th,R}^{p_{2}}e^{-K_{22}\gamma_{\mathrm{th},R}}\\ & +\sum_{k_{1}=0}^{m_{1}-1}\sum_{p_{1}=0}^{k_{1}}\sum_{k_{2}=0}^{m_{2}-1}\sum_{p_{2}=0}^{k_{2}}K_{11}(k_{1},p_{1})K_{21}(k_{2},p_{2})\gamma_{th}^{p_{1}}\gamma_{th,R}^{p_{2}}e^{-K_{12}\gamma_{th}}e^{-K_{22}\gamma_{th,R}}\\ & +\sum_{k_{2}=0}^{m_{2}-1}\sum_{p_{2}=0}^{k_{2}}K_{21}(k_{2},p_{2})\gamma_{th,R}^{p_{2}}e^{-K_{22}\gamma_{th,R}}\\ & -\sum_{k_{2}=0}^{m_{2}-1}\sum_{p_{2}=0}^{k_{2}}\sum_{k=0}^{m_{1}-1}\sum_{p=0}^{k}K_{21}(k_{2},p_{2})K_{41}(k,p)\;\gamma_{th}^{p}\;\gamma_{th,R}^{p_{2}}e^{-K_{12}\gamma_{th}}e^{-K_{22}\gamma_{th,R}}\\ & -\sum_{k_{2}=0}^{m_{2}-1}\sum_{p_{2}=0}^{k_{2}}\sum_{k=0}^{m_{1}-1}\sum_{p=0}^{m_{3}-1}\sum_{q=0}^{p}K_{21}(k_{2},p_{2})K_{42}(k,p,q)\gamma_{th}^{p-q}\gamma_{th,R}^{p_{2}}e^{-C\gamma_{th}}e^{-K_{22}\gamma_{th,R}}\\ & +\sum_{k_{2}=0}^{m_{2}-1}\sum_{p_{2}=0}^{k_{2}}\sum_{k=0}^{m_{1}-1}\sum_{p=0}^{m_{3}-1}\sum_{q=0}^{p}\sum_{j=0}^{k+q}K_{21}(k_{2},p_{2})K_{43}(k,p,q,j)\gamma_{th}^{p-q+j}\gamma_{th,R}^{p_{2}}e^{-K_{12}\gamma_{th}}e^{-K_{22}\gamma_{th,R}}. \end{array}$$

The asymptotic expressions can be derived for some corollary cases as follows:

1. In the case when γ_th,R_→∞, we obtain *I*_2_→1, *I*_3_→0, and the resulting outage probability corresponds to the case without relaying (when only S-D link is present).

(33) $$P_{out}(\gamma_{th})\approx \int_{0}^{\gamma_{th}}f_{\Gamma_{1}}(\gamma_{1})d\gamma_{1}=1-e^{-K_{12}\gamma_{th}}\sum_{k_{1}=0}^{m_{1}-1}\sum_{p_{1}=0}^{k_{1}}K_{11}(k_{1},p_{1})\gamma_{th}^{p_{1}}.$$

2. In the case when γ_th,R_→0 (the reliable S-R link), the instantaneous SNR is obtained as $\gamma (t)=\gamma_{1}(t)+\gamma_{3}(t)$, we obtain *I*_2_→0, *I*_3_→1, and the resulting outage probability is determined with the expression of *I*_4_, i.e.,
  (34) $$\begin{array}{c} P_{out}(\gamma_{th})\approx \int_{0}^{\gamma_{th}}f_{\Gamma_{1}}(\gamma_{1})\int_{0}^{\gamma_{th}-\gamma_{1}}f_{\Gamma_{3}}(\gamma_{3})d\gamma_{3}\;d\gamma_{1}=1-e^{-K_{21}\gamma_{th}}\sum_{k=0}^{m_{1}-1}\sum_{j=0}^{k}K_{41}(k,j)\gamma_{th}^{j}\\ \;\;\;\;\;\;+\sum_{k=0}^{m_{1}-1}\sum_{p=0}^{m_{3}-1}\sum_{q=0}^{p}K_{42}(k,p,q)\gamma_{th}^{p-q}e^{-C\gamma_{th}}-\sum_{k=0}^{m_{1}-1}\sum_{p=0}^{m_{3}-1}\sum_{q=0}^{p}\sum_{j=0}^{k+q}K_{43}(k,p,q,j)\gamma_{th}^{p-q+j}e^{-K_{21}\gamma_{th}}. \end{array}$$

In the case when powerful error correction codes are applied in all communication links, the above expression is a good approximation in the region of very small values for γ_th_.

3. In the case when γ_th,R_ = γ_th_, the derived outage probability reduced to the expression derived in our conference paper [34].

Also, the expressions for simpler DF protocols can be obtained based on the presented analysis, with minor modifications as follows:

1. The analysis for the fixed relaying DF protocol, where it is assumed that the relay retransmits signal even in the case when the instantaneous SNR at the relay is below the threshold, i.e., the outage appears at the destination whenever $\gamma_{2}(t)<\gamma_{\mathrm{th},R}$, i.e.,
  (35) $$P_{out,FR}(\gamma_{th}\left|\gamma_{th,R}\right.)=\int_{0}^{\gamma_{th,R}}f_{\Gamma_{2}}(\gamma_{2})d\gamma_{2}+\int_{\gamma_{th,R}}^{\infty }f_{\Gamma_{2}}(\gamma_{2})d\gamma_{2}\int_{0}^{\gamma_{th}}f_{\Gamma_{1}}(\gamma_{1})\int_{0}^{\gamma_{th}-\gamma_{1}}f_{\Gamma_{3}}(\gamma_{3})d\gamma_{3}\;d\gamma_{1}.$$

Finally, the closed form expression is easily obtained from Equation (32) if the first and third summation (the second and the fourth term) are ignored. This solution represents the closed form solution for the analysis given in [33].

2. The analysis for the simple DF protocol, where it is assumed that the S-D link is blocked [21], can be obtained if we set $\gamma_{1}(t)$ = 0, and therefore
  (36) $$\gamma (t)=\left\{\begin{array}{cc} 0, & \gamma_{2}(t)<\gamma_{\mathrm{th},R},\\ \gamma_{3}(t), & \gamma_{2}(t)\geq \gamma_{\mathrm{th},R}. \end{array}\right.$$

The outage probability is derived through setting ${f_{\Gamma }}_{1}(\gamma_{1})=\delta (\gamma_{1}=0)$, where δ(⋅) denotes the delta function, and it can be determined using the simplified expression as follows:
  (37) $$P_{out,DF}(\gamma_{th}\left|\gamma_{th,R}\right.)=\int_{0}^{\gamma_{th,R}}f_{\Gamma_{2}}(\gamma_{2})d\gamma_{2}+\int_{\gamma_{th,R}}^{\infty }f_{\Gamma_{2}}(\gamma_{2})d\gamma_{2}\int_{0}^{\gamma_{th}}f_{\Gamma_{3}}(\gamma_{3})d\gamma_{3}\;d\gamma_{1}.$$

In this case, the final expression can be obtained in the following form:

(38) $$\begin{array}{l} P_{out,DF}(\gamma_{th}\left|\gamma_{th,R}\right.)=1-\sum_{k_{2}=0}^{m_{2}-1}\sum_{p_{2}=0}^{k_{2}}K_{21}(k_{2},p_{2})\gamma_{th,R}^{p_{2}}e^{-K_{22}\gamma_{\mathrm{th},R}}\\ +\sum_{k_{2}=0}^{m_{2}-1}\sum_{p_{2}=0}^{k_{2}}K_{21}(k_{2},p_{2})\gamma_{th,R}^{p_{2}}e^{-K_{22}\gamma_{th,R}}\left(1-e^{-C\gamma_{th}}\sum_{p_{1}=0}^{m_{3}-1}\frac{1}{p_{1}!}{\left(C\gamma_{th}\right)}^{p_{1}}\right). \end{array}$$

## 4. Ergodic Capacity

In this section, we will derive the ergodic capacity of the land mobile satellite–terrestrial systems with selection relaying. The ergodic capacity represents a maximal long-term rate that can be achieved over a channel with an arbitrarily small probability of error [49]. It is one of the most important performance metrics which is appropriate for applications with no delay requirements, and it is also usually used in the case of fast-fading channels.

The ergodic capacity is defined by the expression

(39) $$C_{e}=E_{\gamma }\left[0.5\log_{2}(1+\gamma )\right],$$

where $E_{\gamma }\left[\cdot \right]$ denotes the operator of statistical averaging over the random variable γ.

The fading statistics of the random variable γ can be easily determined if we notice that the CDF of γ corresponds to the outage probability of the analyzed system when the threshold at the relay receiver is fixed as follows:

(40) $$F_{\Gamma }\left(\gamma_{th}\left|\gamma_{th,R}\right.\right)=P_{out}\left(\gamma_{th}\left|\gamma_{th,R}\right.\right).$$

Based on the well-known relation for the distributions of the continuous random variables, the corresponding PDF is obtained as

(41) $$f_{\Gamma }(\gamma \left|\gamma_{th,R}\right.)=\frac{\partial F_{\Gamma }(\gamma \left|\gamma_{th,R}\right.)}{\partial \gamma }.$$

Using the fact that the outage probability is represented in the form of a finite summation of the polynomial–exponential terms, the final closed-form expression for the PDF of the SNR at the output of the MRC combiner is derived as

(42) $$\begin{array}{cc} f_{\Gamma } & (\gamma \left|\gamma_{th,R}\right.)=-\sum_{k_{1}=0}^{m_{1}-1}\sum_{p_{1}=0}^{k}K_{11}(k_{1},p_{1})P(p_{1},K_{12})\;\\ & +\sum_{k_{1}=0}^{m_{1}-1}\sum_{p_{1}=0}^{k}\sum_{k_{2}=0}^{m_{2}-1}\sum_{p_{2}=0}^{k}K_{21}(k_{2},p_{2})K_{11}(k_{1},p_{1})P(p_{1},K_{12})\gamma_{th,R}^{p_{2}}e^{-K_{22}\gamma_{th,R}}\\ & -\sum_{k_{2}=0}^{m_{2}-1}\sum_{p_{2}=0}^{k_{2}}\sum_{k=0}^{m_{1}-1}\sum_{p=0}^{k}K_{21}(k_{2},p_{2})K_{41}(k,p)P(p,K_{12})\gamma_{th,R}^{p_{2}}e^{-K_{22}\gamma_{th,R}}\\ & -\sum_{k_{2}=0}^{m_{2}-1}\sum_{p_{2}=0}^{k_{2}}\sum_{k=0}^{m_{1}-1}\sum_{p=0}^{m_{3}-1}\sum_{q=0}^{p}K_{21}(k_{2},p_{2})K_{42}(k,p,q)P(p-q,C)\gamma_{th,R}^{p_{2}}e^{-K_{22}\gamma_{th,R}}\\ & +\sum_{k_{2}=0}^{m_{2}-1}\sum_{p_{2}=0}^{k_{2}}\sum_{k=0}^{m_{1}-1}\sum_{p=0}^{m_{3}-1}\sum_{q=0}^{p}\sum_{j=0}^{k+q}K_{21}(k_{2},p_{2})K_{43}(k,p,q,j)P(p-q+j,K_{12})\gamma_{th,R}^{p_{2}}e^{-K_{22}\gamma_{th,R}}. \end{array}$$

In the above expression, *P*(*m*,*u*) represents a shorter notation for the polynomial–exponential term obtained using the derivation of the expression $\gamma^{m}e^{-u\gamma }$, i.e.,

(43) $$P(m,u)=\frac{\partial }{\partial \gamma }(\gamma^{m}e^{-u\gamma })=m\gamma^{m-1}e^{-u\gamma }-u\gamma^{m}e^{-u\gamma }.$$

In the case when γ_th,R_→∞, only the first term remains, and this corresponds to the system with the S-D link only. If γ_th,R_→0, the PDF can be simplified to the expression that can be easily derived by combining (34) and (41).

When the PDF is known, Equation (39) can be expressed as follows:

(44) $$C_{e}=0.5\int_{0}^{\infty }\log_{2}(1+\gamma )f_{\Gamma }(\gamma )d\gamma .$$

The final closed-form expression for the ergodic capacity of the analyzed system is derived as

(45) $$\begin{array}{cc} C_{e}(\gamma ) & =-0.5\sum_{k_{1}=0}^{m_{1}-1}\sum_{p_{1}=0}^{k}K_{11}(k_{1},p_{1})Q(p_{1},K_{12})\\ & +0.5\sum_{k_{1}=0}^{m_{1}-1}\sum_{p_{1}=0}^{k}\sum_{k_{2}=0}^{m_{2}-1}\sum_{p_{2}=0}^{k}K_{11}(k_{1},p_{1})K_{21}(k_{2},p_{2})Q(p_{1},K_{12})\gamma_{th,R}^{p_{2}}e^{-K_{22}\gamma_{th,R}}\\ & -0.5\sum_{k_{2}=0}^{m_{2}-1}\sum_{p_{2}=0}^{k_{2}}\sum_{k=0}^{m_{1}-1}\sum_{p=0}^{k}K_{21}(k_{2},p_{2})K_{41}(k,p)Q(p,K_{12})\gamma_{th,R}^{p_{2}}e^{-K_{22}\gamma_{th,R}}\\ & -0.5\sum_{k_{2}=0}^{m_{2}-1}\sum_{p_{2}=0}^{k_{2}}\sum_{k=0}^{m_{1}-1}\sum_{p=0}^{m_{3}-1}\sum_{q=0}^{p}K_{21}(k_{2},p_{2})K_{42}(k,p,q)Q(p-q,C)\gamma_{th,R}^{p_{2}}e^{-K_{22}\gamma_{th,R}}\\ & +0.5\sum_{k_{2}=0}^{m_{2}-1}\sum_{p_{2}=0}^{k_{2}}\sum_{k=0}^{m_{1}-1}\sum_{p=0}^{m_{3}-1}\sum_{q=0}^{p}\sum_{j=0}^{k+q}K_{21}(k_{2},p_{2})K_{43}(k,p,q,j)Q(p-q+j,K_{12})\gamma_{th,R}^{p_{2}}e^{-K_{22}\gamma_{th,R}}, \end{array}$$

where

(46) $$\begin{array}{c} Q(m,u)=\int_{0}^{\infty }\log_{2}(1+\gamma )P(m,u)d\gamma \\ \;\;\;=\frac{m}{\ln(2)}\int_{0}^{\infty }\ln(1+\gamma )\gamma^{m-1}e^{-u\gamma }d\gamma -\frac{u}{\ln(2)}\int_{0}^{\infty }\ln(1+\gamma )\gamma^{(m+1)-1}e^{-u\gamma }d\gamma . \end{array}$$

Using the identity [50]

(47) $$\int_{0}^{\infty }\ln(1+\gamma )\gamma^{m-1}e^{-u\gamma }d\gamma =(m-1)!e^{u}\sum_{k=1}^{m}\frac{\Gamma (-m+k,u)}{u^{k}},$$

term *Q*(*m*,*u*) can be represented in the closed form as a finite summation of upper incomplete Gamma functions.

(48) $$Q(m,u)=\frac{me^{u}(m-1)!}{\ln(2)}\sum_{k=1}^{m}\frac{\Gamma (-m+k,u)}{u^{k}}-\frac{ue^{u}m!}{\ln(2)}\sum_{k=1}^{m+1}\frac{\Gamma (-m-1+k,u)}{u^{k}},$$

where the upper incomplete Gamma function with the negative integer argument *s* can be represented in the polynomial form [51].

(49) $$\Gamma (-n,z)=\frac{1}{n!}\left(\frac{e^{-z}}{z^{n}}\sum_{k=0}^{n-1}{\left(-1\right)}^{k}(n-k-1)!z^{k}+{\left(-1\right)}^{n}\Gamma (0,z)\right).$$

## 5. Numerical Results

In this section, we present the numerical results of the outage probability, outage capacity, and ergodic capacity analyzed in previous chapters. The average SNR at every particular link is determined based on the typical parameters of the practical systems, and the time-varying channel gains are generated using a simulator that correctly captures the first- and second-order statistics of the random processes related to the multipath and shadowing effects.

Numerical results will be presented for an LEO satellite system, where the average SNR at the receiver is determined using [11,37]

γ_1_ [dB] = *EIRP* [dB] − *n_SD_* × 10log10(4π*d_SD_f*_0_/*c*) − *L*_A_ [dB] + *G* [dB] − 10log10(*kT_S_*B), (50)

where EIRP denotes the effective isotropic radiated power, *L_A_* denotes atmospheric losses due to oxygen and water as well as other losses (polarization mismatch, antenna misalignment), and G denotes the antenna gain at the receiver. The second term at the right side of the above equation corresponds to free-space path loss (FSPL), where *d_SD_* denotes the distance between the satellite and receiver at the destination, *n_SD_* denotes the corresponding path loss factor, *f*_0_ denotes the carrier frequency at the satellite–terrestrial link, and *c* = 3 × 10^8^ m/s denotes the speed of light. The last term in (50) corresponds to the noise level, where *k* = 1.38 × 10^−23^ J/K denotes the Boltzmann constant, *T_S_* denotes the temperature of the system, and *B* denotes the channel bandwidth.

We can rewrite Equation (44) in the logarithm representation of Equation (3) as follows:

γ_1_ [dB] = *P_S_* [dB] − *n_SD_* × 10log_10_(*d_SD_*) − 10log10(*σ*^2^), (51)

where variable *P_S_* takes into account the same power-related parameters for all system users:

*P_S_* [dB] = EIRP [dB] − *n_SD_* × 10log_10_(4π*f*_0_/*c*) − *L*_A_[dB] + G [dB], (52)

the second term takes into account the variable parameters of FSPL (distance and path loss factor), and the third factor corresponds to the power of the noise at the receiver.

The satellite–terrestrial downlink operates in the Ku-band, and typical values for EIRP, *L_A_*, *f*_0_, *G*, *T_S_*, and *B* for active LEO satellite systems can be found in [5,11]. Due to the relatively small diameter of the beam (*L* << *H*), it can be assumed that the elevation angles at both the destination and relay satisfy the condition θ_R_ = θ_D_ = θ, and the corresponding distances and path loss factors are approximately the same, i.e., *d_SD_* ≈ *d_SR_*, *n*_SD_ ≈ *n*_SR_, and therefore $\bar{\gamma }={\bar{\gamma }}_{i},\;i=1,2.$ The terrestrial link operates in the urban environment, with a higher path loss factor, where *n_RD_* = 4 [52]. If it is not differently stated, we also assume that the *P_R_* is adjusted to provide approximately the same average SNR in all channels (i.e., ${\bar{\gamma }}_{i}=\bar{\gamma },\;i=1,2,3$), and, for the parameters presented in Table 2, we obtain that $\bar{\gamma }=13.2\;dB.$

**Figure 3:**
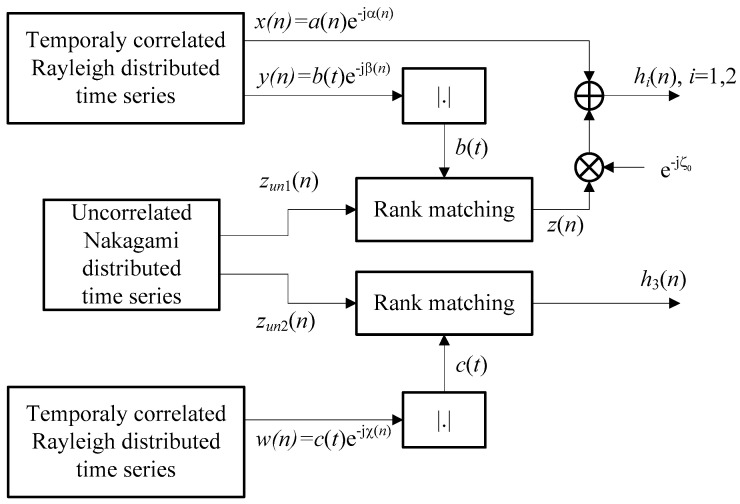
The simulator of the Nakagami-*m* and shadowed Rice channel gains with desired first- and second-order statistics.

Applying the method illustrated in Figure 3, we have generated the temporally correlated time series that corresponds to the channel gains of the satellite–terrestrial links and the channel gain of the terrestrial link as follows:

- We apply the method based on autoregressive models [53] to generate the time series *x*(*n*) that describes the multipath component. It corresponds to the complex Gaussian random process with a Rayleigh distributed envelope. In the case of isotropic scattering, the normalized autocorrelation function is given by $R_{r}(\tau )=J_{0}(2\pi f_{Dm}\tau )$, where *f_Dm_* denotes the maximum Doppler shift for the multipath component (we assume *f_Dm_* = 100 Hz).
- The first step is repeated to generate a time series *y*(*n*), independent from *x*(*n*), with the Rayleigh distributed envelope and ACF $R_{s}(\tau )=J_{0}(2\pi f_{Ds}\tau )$, where *f_Ds_* denotes the maximum Doppler shift for the shadowing (which is usually *f_Ds_ << f_Dm_*, while in our simulations, we chose *f_Ds_* = 1 Hz [54]).
- Based on the rejection/acceptance technique described in [55,56], we have generated a temporally uncorrelated time series *z_un_*_1_(*n*) with Nakagami distribution. The rank matching method described in [47] is applied to reorder the samples in that process according to the previously generated reference *y*(*n*). The resulting time series *z*(*n*) corresponds to the time-varying LOS component that has an envelope with Nakagami distribution (as in *z_un_*_1_(*n*)) and a normalized ACF $R_{s}(\tau )=J_{0}(2\pi f_{Ds}\tau )$(as in *y*(*n*)).
- Two previous steps are repeated to generate *w*(*n*) with a Rayleigh distributed envelope and the maximum Doppler shift *f_Dt_*. It was combined with the temporally uncorrelated time series *z_un_*_2_(*n*), resulting in the time series *h*_3_(*n*) with a Nakagami distributed envelope and a normalized ACF, where $R_{t}(\tau )=J_{0}(2\pi f_{Dt}\tau )$. This corresponds to the channel gain of the terrestrial R-D link.
- Channel gains for any satellite–terrestrial link (S-D or S-R) are obtained through the expression *h*(*n*) = *x*(*n*) + *w*(*n*).

**Figure 4:**
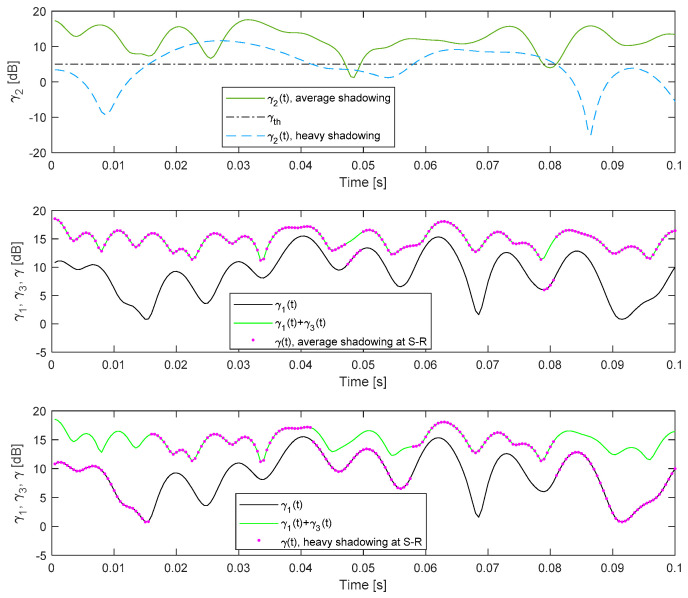
The discrete waveforms of the instantaneous SNRs at the S-R link, γ_2_(*n*), at the S-D link, γ_1_(*n*), γ_3_(*n*), and at the output of the MRC receiver at the destination, γ(*n*).

Figure 4 shows the instantaneous SNRs at the receiver in the relay (S-R link), at the receiver in the destination when the relay is not present (S-D link), and at the output of the MRC combiner at the destination when cooperative relaying is applied. The simulator for the shadowed Rice fading is applied to determine the samples of the channel gains in the S-D and S-R link, the simulator for the Nakagami fading is applied to determine samples of the channel gain in the R-D link, and the corresponding samples of the time-varying SNR values are obtained using the identity (2). Finally, the *n*-th sample of the SNR waveform at the output of the MRC combiner is determined using

(53) $$\gamma (n)=\left\{\begin{array}{cc} {\bar{\gamma }}_{1}{\left|h_{1}(n)\right|}^{2}, & {\bar{\gamma }}_{2}{\left|h_{2}(n)\right|}^{2}<\gamma_{th,R},\\ {\bar{\gamma }}_{1}{\left|h_{1}(n)\right|}^{2}+{\bar{\gamma }}_{3}{\left|h_{3}(n)\right|}^{2}, & {\bar{\gamma }}_{2}{\left|h_{2}(n)\right|}^{2}\geq \gamma_{th,R}. \end{array}\right.$$

Typical channel parameters for the satellite–terrestrial channel are determined in [12], and these values are presented in Table 3, where the fading parameter *m* is rounded to the nearest integer value. The time series that correspond to the SNR waveforms are shown in Figure 4 for average shadowing in the S-D link, the typical parameters for the terrestrial R-D link (*m*_3_ = 5, Ω_3_ = 1), and heavy or average shadowing at the S-R link.

It is obvious that the propagation conditions in the S-R link have an important impact on the instantaneous SNR at the output of the MRC combiner. In the case where γ_th,R_ = 5 dB, and an average shadowing scenario at the S-R link, the corresponding SNR will usually be above the SNR threshold, and we can notice that γ(*n*) ≈ γ_1_(*n*) + γ_3_(*n*). However, in the case of heavy shadowing at the S-R link, the impact of the relaying is less significant, and the output SNR values are similar to the SNR values at the S-D link.

The numerical results presented in Figure 4 clearly illustrate that relaying will be more effective for lower values of γ_th,R_. Furthermore, we can notice that every particular value of the threshold at the relay results in a different waveform of the SNR at the output of the MRC combiner. Therefore, statistics of the SNR at the destination receiver depend on the threshold applied at the relay receiver.

**Figure 5:**
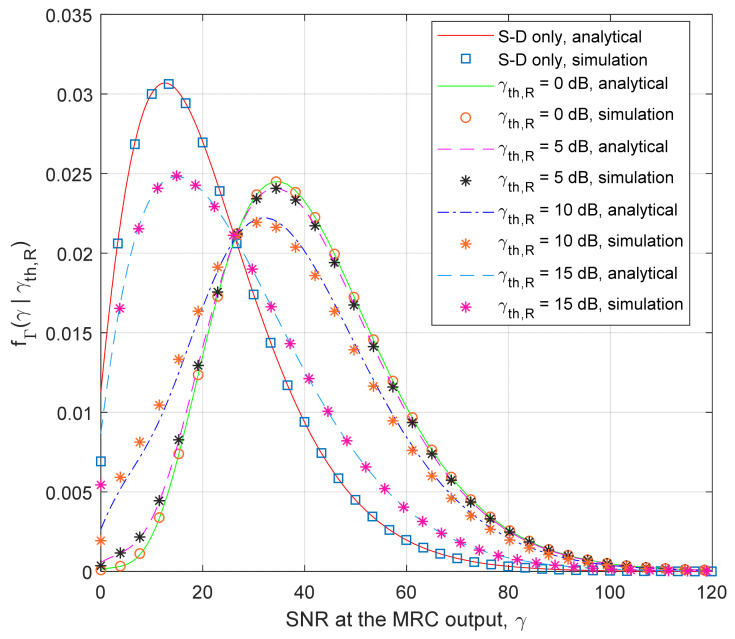
PDF of the signal–noise at the MRC output for various thresholds at the relay.

The PDF curves for the SNR at the MRC output are illustrated in Figure 5, these demonstrate the dependence on the threshold γ_th,R_. The theoretical results are obtained using Equation (42), and the simulation results are estimated based on *N* = 10^7^ samples for the case of the average shadowing at the S-D and S-R channel, and the fading parameter in the R-D channel has the value *m*_3_ = 5. In accordance with the expectations, lower values of the threshold at the relay result in distributions with larger mean values. For smaller values of γ_th,R_, the probability that the SNR at the MRC output in the destination belongs to the range of large values (e.g., larger than 15 dB, or in absolute values γ > 31.63) increases. Therefore, we can expect that the outage probability will be reduced, and the system capacity will be increased if the SNR threshold at the relay has smaller values.

However, the SNR threshold is mostly determined using the applied modulation and error control techniques in the implemented communication protocol, as well as the required quality of service. In most cases, it is not easy to design practical communications techniques that provide reliable transmissions for γ_th,R_ < 5 dB.

In most cases, it is reasonable to assume that the SNR threshold has the same value in the receiver implemented at the destination and in the receiver implemented at the relay. This assumption will be used to calculate the outage probability and the outage capacity. When the ergodic capacity is calculated, the instantaneous capacity is averaged using the corresponding PDF, and the threshold at the end receiver (located at the destination) is not used. However, in this system, the PDF of the SNR at the destination depends on the threshold at the relay. Therefore, the ergodic capacity will also be determined for the various values of the threshold γ_th,R._

It is interesting to verify the statement that the temporal properties of the channel gains *h*_1_(*t*) and *h*_2_(*t*) are mostly determined using the ACF of the corresponding scattering component. The discrete representation of the instantaneous channel gain in the *i*-th channel (*i* = 1, 2), given by *h_i_*(*k*) = *x_i_*(*n*) + *z_i_*(*n*), has the following discrete autocorrelation function:

(54) $$R_{h,i}(k)=E\left[\left(x_{i}(n)+z_{i}(n)e^{j\varsigma }\right)\left({x_{i}}^{*}(n+k)+z_{i}(n+k)e^{-j\varsigma }\right)\right],$$

which can be simplified using the properties of the mathematical expectation operator

(55) $$R_{h,i}(k)=E\left[x_{i}(n){x_{i}}^{*}(n)\right]+e^{-j\varsigma }E\left[x_{i}(n)z_{i}(n+k)\right]+e^{j\varsigma }E\left[z_{i}(n){x_{i}}^{*}(n+k)\right]+E\left[z_{i}(n)z_{i}(n+k)\right].$$

Series *x_i_*(*n*) and *z*_i_(*n*) are mutually independent, as the scattered and LOS components are assumed to be statistically independent in the shadowed Rice channel model [12]. Therefore, the second and the third expectation operators are equal to zero. The first expectation corresponds to the ACF of the complex time series *x_i_*(*n*), denoted by *R_x_*_,*i*_(*k*), and the fourth expectation corresponds to the ACF of the real-valued series *z*(*n*), denoted by *R_z_*_,*i*_(*k*).

Based on the theory presented in Section 4, the scattering component of the *i*-th channel gain with the Rayleigh distribution and the average power 2*b*_0,*i*_, estimated from the generated time series *x*(*n*), should have the following discrete autocorrelation function:

(56) $$R_{x,i}(k)=2b_{0,i}J_{0}(2\pi f_{Dm}kT_{S}),$$

and the corresponding LOS component with the Nakagami-*m* distribution and the average power Ω*_i_*, estimated from the generated time series *z*(*n*), should have the discrete ACF.

(57) Rz,i(k)=ΩiΓ2(mi+1/2)miΓ2(mi)F12−12,−12;mi;J02(2πfDskTS).

**Figure 6:**
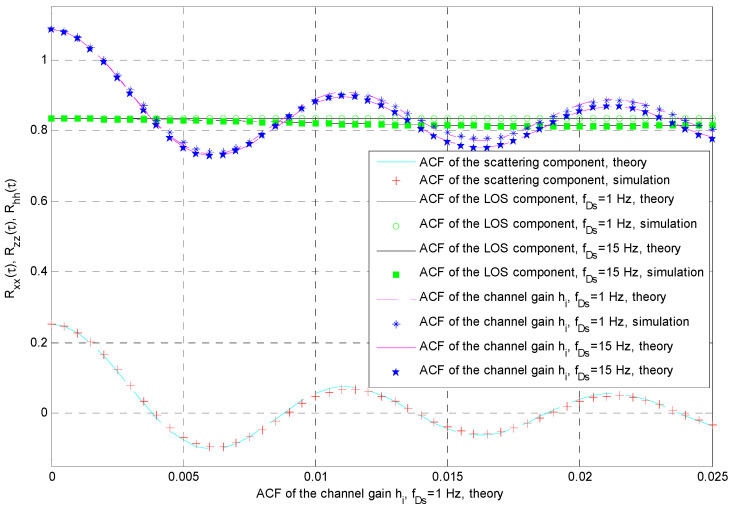
ACF of the channel gain, average shadowing, *f_Dm_* = 100 Hz, *f_Ds_* = 1 Hz, and *f_Ds_* = 15 Hz.

Finally, the discrete ACF of the channel gain in the *i*-th channel can be calculated by combining two previous expressions, i.e., *R_h_*_,*i*_(*k*) = *R_x_*_,*i*_(*k*) + *R_z_*_,*i*_(*k*). The accuracy of the simulator is demonstrated in Figure 6 for the case of the average shadowing scenario, previously determined using Doppler shifts (*f_Dm_* = 100 Hz, *f_Ds_* = 1 Hz), and for the case where *f_Ds_* = 15 Hz. The simulation results correspond very well with the theoretical expressions for the ACF. If the LOS varies slowly, the corresponding ACF *R_z_*_,*i*_(*k*) is almost constant for the observed lags, and the shape of *R_h_*_,*i*_(*k*) is mostly determined using the shape of *R_x_*_,*i*_(*k*). It is easy to verify that the normalized autocovariance functions of time series *h_i_*(*n*) and *x_i_*(*n*), denoted, respectively, by *C_h_*_,*i*_(*k*) and *C_x_*_,*i*_(*k*), would satisfy the relation *C_h_*_,*i*_(*k*) ≈ *C_x_*_,*i*_(*k*).

It is convenient to compare the instantaneous capacity of the proposed cooperative relaying system with the system that uses the S-D link only and the same parameters (referent system).

The instantaneous capacity of the described system with cooperative relaying is given by

(58) $$C(t)=0.5\log_{2}\left(1+\gamma (t)\right),$$

while the instantaneous capacity of the referent system is given by

(59) $$C_{\mathrm{ref}}(t)=\log_{2}\left(1+\gamma_{1}(t)\right).$$

The system without relaying is more efficient in the usage of the available bandwidth, but it usually operates with a smaller SNR at the destination, and it is not obvious if the effects of the relaying will be positive for any combination of system and channel parameters. An alternative definition of the outage probability is as follows:

(60) $$P_{out}(C_{th})=\mathrm{Pr}\left\{C(t)\leq C_{th}\right\},$$

where *C_th_* = log_2_(1 + γ*_th_*) denotes a predefined capacity threshold.

**Figure 7:**
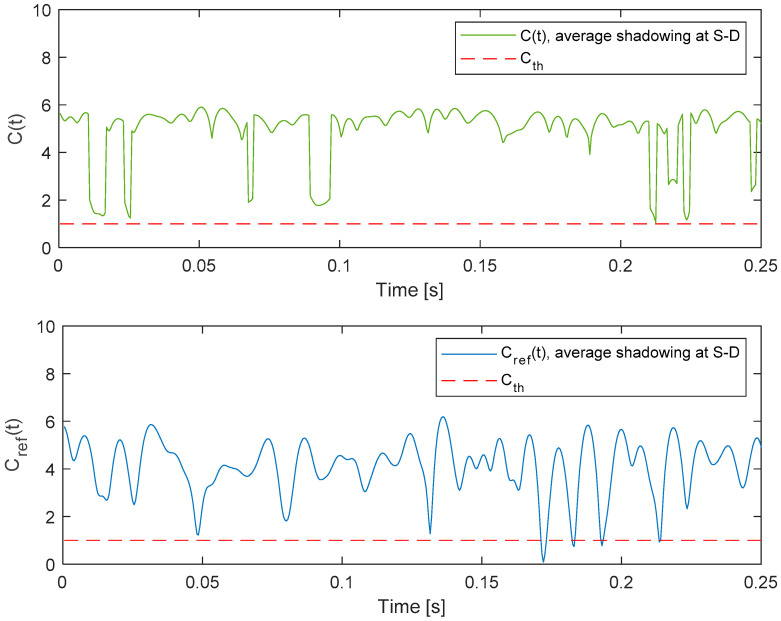
Instantaneous capacity of the system with relaying *C*(*t*) and the referent system *C*_ref_(*t*).

The time series that correspond to the instantaneous capacity of the system with relaying and the referent system are shown in Figure 7. The waveforms correspond to the average shadowing at both satellite–terrestrial links (S-D and S-R) and the average propagation conditions at the terrestrial R-D link (*m*_3_ = 5, Ω_3_ = 1). It is interesting to note that the average value of the capacity is larger in the referent system, as the benefit of MRC cannot compensate for the inefficient use of the bandwidth due to the relaying. However, during the observed interval, the instantaneous capacity of the system with relaying does not decrease below the threshold *C_th_* = 1 bit/s/Hz, contrary to the instantaneous capacity of the referent system. This indicates that the ergodic capacity of the system with relaying is lower when compared to the referent system, while the outage capacity of the system with relaying can be larger when compared to the referent system for chosen system parameters and this particular capacity threshold.

It was shown that the instantaneous capacity of the analyzed system can be obtained through Equation (58), and it is interesting to check if it is possible to determine the normalized instantaneous SNR if the system without relaying (with more efficient use of the bandwidth) could provide the same capacity in every particular instant.

(61) $$C(t)=\log_{2}\left(1+\gamma_{norm}(t)\right).$$

The relation between the SNR at the output of the MRC combiner and the normalized SNR that could result in the same instantaneous capacity in the system without relaying can be found based on the following identity:

(62) $$0.5\log_{2}(1+\gamma (t))=\log_{2}(1+\gamma_{\mathrm{norm}}(t)),$$

and it is easy to find the relation

(63) $$\gamma (t)=\gamma_{\mathrm{norm}}^{2}(t)+2\gamma_{\mathrm{norm}}(t).$$

Based on the above equation, we can notice that the system with relaying achieves a higher capacity when compared to the referent system (where only the S-D link is present) in the time instants *t* where it is satisfied as follows: $\gamma_{\mathrm{norm}}(t)>\gamma_{1}(t).$ The comparison of the analyzed and referent system can be performed for the same normalized threshold $\gamma_{\mathrm{th},\mathrm{norm}}$ that satisfies the relation $\gamma_{\mathrm{th}}=\gamma_{\mathrm{th},\mathrm{norm}}^{2}+2\gamma_{\mathrm{th},\mathrm{norm}}$ [48]. The outage probabilities of the system with relaying and the referent system are, respectively, given by

(64) $$P_{out}(\gamma_{\mathrm{th},\mathrm{norm}})=\mathrm{Pr}\left(\gamma \leq \gamma_{\mathrm{th},\mathrm{norm}}^{2}+2\gamma_{\mathrm{th},\mathrm{norm}}\right),$$

(65) $$P_{out,ref}(\gamma_{\mathrm{th},\mathrm{norm}})=\mathrm{Pr}\left(\gamma_{1}\leq \gamma_{\mathrm{th},\mathrm{norm}}\right).$$

**Figure 8:**
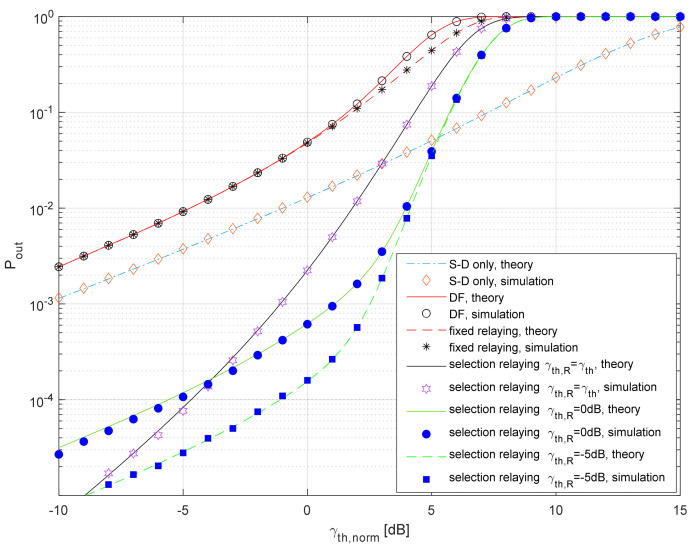
Outage probability vs. normalized SNR threshold for various protocols.

In Figure 8, the outage probability of the analyzed system is presented for the average shadowing at the satellite–terrestrial link and Nakagami-*m* fading with *m*_3_ = 5 at the terrestrial link. Also, we provide the comparison of three DF-based protocols as follows:

- simple DF protocol, for the case where the S-D link is blocked, as a typical scenario for HSTRNs, previously analyzed in [21];
- fixed relaying protocol, i.e., the DF protocol applied for the case where the S-D link is present, the relay always retransmits the signal, and MRC is applied at the destination was analyzed in [33];
- the selection relaying protocol, analyzed in this paper, where the S-D link is present, the relay retransmits the signal only if the instantaneous SNR at the relay is larger than γ_R_, and MRC is applied at the destination [33,34].

In the available literature, it is usually assumed that the threshold at the relay γ_th,R_ is equal to the threshold at the destination γ_th_. Taking into the account that the same modulation and coding scheme is used in all communication links, this assumption is reasonable in the case where the decoding is always successful if the SNR is above the threshold, and always unsuccessful if the SNR is below the threshold.

Although the error probability decreases with the increasing average SNR, in the more realistic scenario, it has a finite value even when the instantaneous SNR is above the threshold. In such a case, an optimal value for the threshold at the relay can be determined for every particular value of the average SNR by minimizing the error probability at the destination. This procedure was explained in paper [57] for the case of terrestrial cooperative systems with Nakagami-*m* propagation and binary phase shift keying (BPSK) modulation. The optimized value γ_th,R_ depends on the applied modulation and coding scheme, as well as the SNR statistics at the communication links. Although analysis is out of the scope of this paper, we present the numerical results for the outage probability for the two following simplified cases:

- γ_th,R_ = γ_th_, as assumed in most of the papers;
- value γ_th,R_ is fixed, and *P_out_* is given for a typical range of γ_D_.

The theoretical results are obtained using Equations (32)–(38), and the Monte Carlo simulation results are estimated based on *N* = 10^7^ samples and the basic principles of communication system simulations [58]. It can be observed that a typical DF protocol [21], applied when the S-R link is blocked (*P_out_* given in Equation (38)), exhibits inferior performance when compared to the referent system, with *P_out_* given in Equation (33). When DF is combined with MRC, a minor improvement is visible if the relay always retransmits the received signal [33]. The selection relaying has a potential to further improve the performance of the satellite–terrestrial network [33], and the closed-form expression for the outage probability was derived in our conference paper [34] for the case where γ_th,R_ = γ_th_. In this paper, we have derived a more general expression for the outage probability, valid for γ_th,R_ ≠ γ_th_, when the satellite–terrestrial links undergo shadowed Rice fading, and propagation in terrestrial links can be described using Nakagami-*m* statistics. Although the scenario with the fixed γ_th,R_ is simplified, it can be noticed that additional performance improvements are possible if γ_th,R_ < γ_th_. If γ_th,R_→0 and γ_th_→0, the performances of the system with highly reliable links can be estimated from the simplified expression (34). This can be used as a guideline for the further optimization of parameter γ_th,R_, based on the procedure presented in [57].

**Figure 9:**
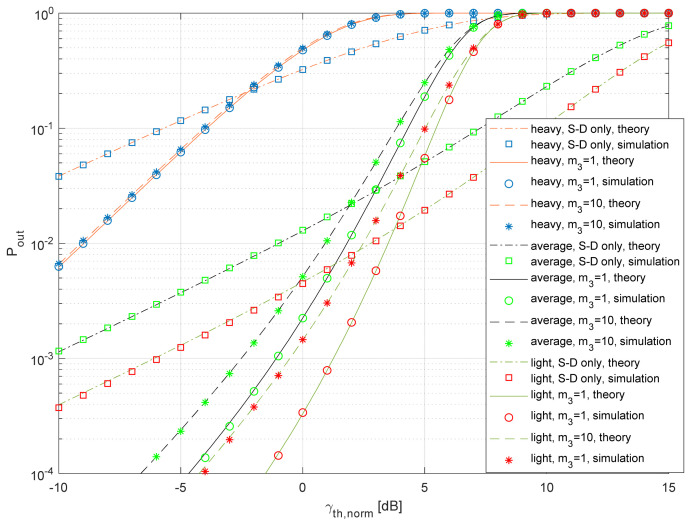
Outage probability vs. normalized SNR threshold for various propagation scenarios.

In Figure 9, we show the outage probability for the practically important cases when the SNR thresholds at the relay and destination are the same, as MRC diversity can be applied at the destination if the same modulation and coding schemes are applied both in the S-D and R-D links. Also, we assumed that both satellite–terrestrial links (S-D and S-R) experience the same level of shadowing. In accordance with the expectations, outage probability decreases for better propagation conditions in satellite–terrestrial links S-D and S-R, as well as for the increased value of the fading factor *m*_3_ in the terrestrial link R-D. It is interesting to notice that the referent system (where only the S-D link is present) outperforms the system by relaying for larger threshold values. This can be explained using Figure 6, where, for the presented samples, we estimate that *P*_out_(*C*_th_) = 1 and *P*_out,ref_(*C*_th_) < 1 if *C*_th_ = 5. The effect of relaying is more visible for the light shadowing in satellite–terrestrial links, and, in such a case, the outage probability is lower when compared to the referent system, even for high threshold levels. Furthermore, it can be noticed that the outage probability of the system is more sensitive to the quality of the R-D link if there is light shadowing in the S-D link and S-R link.

**Figure 10:**
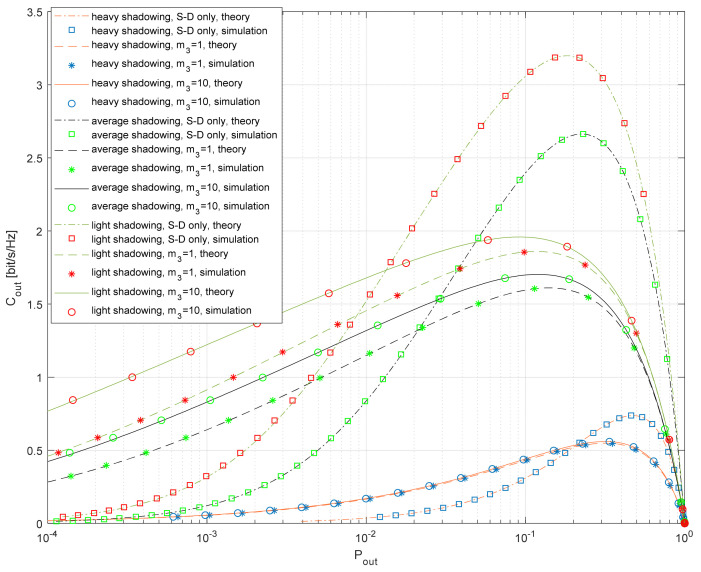
Outage capacity vs. outage probability for various propagation scenarios.

The dependence of the outage capacity on the outage probability is shown in Figure 10. It is well known that the outage capacity has a low value when the outage probability is large (the capacity threshold is not achieved), as well as for small outage probabilities (the capacity at the threshold is too small). Therefore, there is always an optimal value of the outage probability that maximizes the outage capacity. Light shadowing in the S-D and S-R links results in the largest values of *C_out_* for any value of the target outage probability when compared to the other propagation scenarios. Furthermore, the impact of the fading factor in the R-D link is largest for the light shadowing. The system with relaying outperforms the referent system for lower outage probabilities only. This effect is more noticeable in the case of light shadowing in satellite–terrestrial links, where the referent system provides larger *C_out_* in a wide range of practically important outage probabilities.

**Figure 11:**
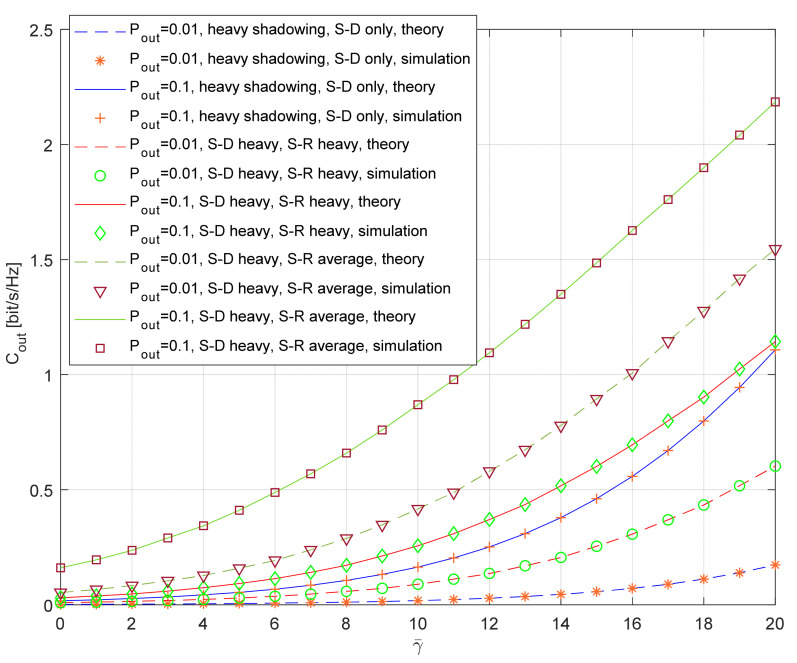
Outage capacity for heavy shadowing in the S-D channel and various outage probabilities.

The dependence of the outage capacity on the average SNR for the various outage probabilities is shown in Figure 11. The theoretical results for the outage capacity are obtained through combining Equations (17) and (32). The simulation results are obtained using the generated waveforms for the SNRs in all channels, with the length *N* = 10^7^. The instantaneous capacity for the system with relaying and the referent system is determined using Equations (15) and (16), respectively. The outage capacity is estimated through comparing the instantaneous capacity with the corresponding threshold.

We assume the most critical propagation scenario with the heavy shadowing at the S-D link, which results in the worst system performance. The numerical results are presented for the typical elevation angle θ = 60°, the parameters of the Nakagami fading at the R-D link are *m*_3_ = 5 and Ω_3_ = 1, and the transmitted power at the relay is adjusted to satisfy the equation $P_{R}=P_{S}\times d_{RD}^{n_{RD}}/d_{SD}^{n_{SR}},$ and, therefore, $\bar{\gamma }={\bar{\gamma }}_{i},\;i=1,2,3.$ The presented numerical results indicate that relaying increases the outage capacity of the satellite–terrestrial system for typical values of the average SNR, although the system with relaying is spectrally less efficient. If the heavy shadowing is present both in the S-D and the S-R link, the capacity gain due to relaying is more significant for larger values of the average SNR and smaller values of the outage probability (if $\bar{\gamma }=20\;\mathrm{dB}$, outage capacity *C_out_* = 3.48*C_out_*_,*ref*_ for *P_out_* = 0.01, and *C_out_* = 1.03*C_out_*_,*ref*_ for *P_out_* = 0.1).

We have also considered another interesting propagation scenario, where heavy shadowing is present in the S-D link and the average shadowing is present in the S-R link. As the typical diameter of the beam is *L* = 25 km, the destination and the relay can be placed at positions with different shadowing scenarios, and it is reasonable to assume that the position of the fixed relay is chosen more optimally. As expected, in the case of average shadowing at the S-R link, the outage capacity is additionally increased. Although this gain is larger for *P_out_* = 0.01, it is also significant for *P_out_* = 0.1.

**Figure 12:**
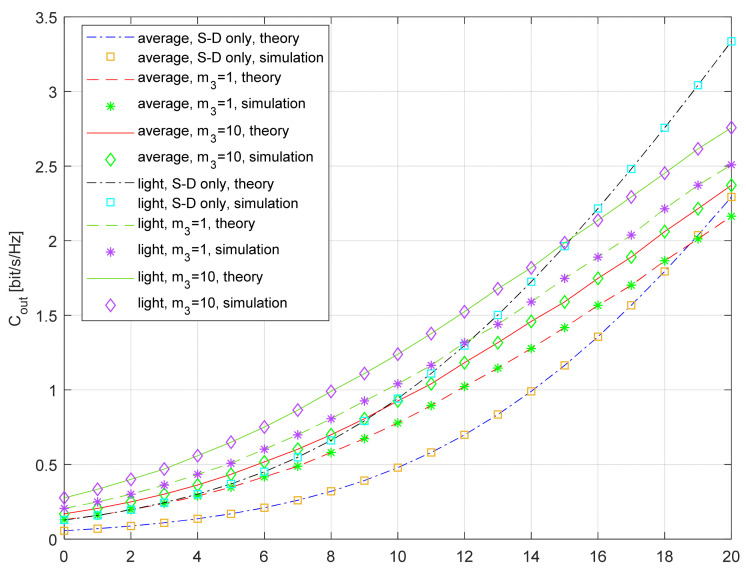
Outage capacity vs. average SNR; various propagation conditions, *P_out_* = 0.01.

The dependence of the outage capacity on the average SNR for the various fading parameters at the R-D link is shown in Figure 12. The numerical results are presented for the fixed outage probability *P_out_* = 0.01, and numerical results are obtained using the time series with *N* = 10^7^ samples. The two following scenarios are considered—the first one when average shadowing is present both at the S-D and the S-R link, and the second one when light shadowing is present at both links. The numerical results are presented for two values of the Nakagami-*m* fading parameter at the R-D link, namely *m*_3_ = 1 and *m*_3_ = 10. The increase in the outage capacity due to the applied selection relaying is visible in a wide range of average SNRs, both for the average and light shadowing.

In the case of light shadowing at the S-D and S-R links and when the Nakagami-*m* fading parameter at the R-D link is equal to *m*_3_ = 1, the outage capacity for the system with relaying is greater than the outage capacity of the referent system (S-D only) in the range $\bar{\gamma }<12\;\mathrm{dB}$. For the same propagation conditions at the communication links between satellite and terrestrial receivers, but for the increased fading parameter at the terrestrial link (*m*_3_ = 10), the improvement due to relaying is visible in the range $\bar{\gamma }<15\;\mathrm{dB}$.

In the case of average shadowing at the S-D and S-R links and *m*_3_ = 1, the outage capacity for the system with relaying is greater than the outage capacity of the referent system in the range $\bar{\gamma }<18.5\;\mathrm{dB}$. A greater value of the Nakagami-*m* fading parameter in the terrestrial channel leads to a higher outage capacity. If *m*_3_ = 10, for the same propagation conditions at the communication links between the satellite and terrestrial receivers, the improvement due to relaying is visible in the whole analyzed range $\bar{\gamma }<20\;\mathrm{dB}$.

In general, the outage capacity improvement is more significant in the range of a low average SNR and for larger values of the Nakagami-*m* fading parameter at the terrestrial communication link. Although the light shadowing results in larger values of the outage capacity, the improvement due to the relaying is less in this propagation scenario.

Finally, when observing Figure 10, Figure 11 and Figure 12, it can be concluded that the outage capacity can be increased with the support of the selection relaying applied at the terrestrial user, and the capacity increase is most significant in the worst propagation conditions (heavy or average shadowing, a low average SNR). When the required outage probability is low, the increase is more significant, and it is noticeable even for light shadowing and high values of the average SNR.

**Figure 13:**
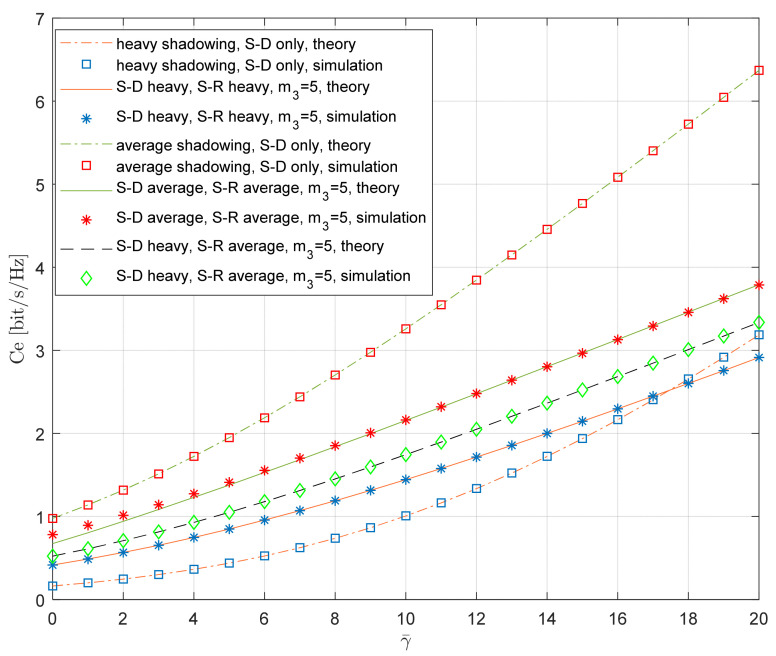
Ergodic capacity vs. average SNR; various propagation conditions.

The curves in Figure 13 show the dependence of the ergodic capacity on the average SNR. The theoretical results are obtained using Equation (45), and the simulation results are obtained using the Monte Carlo simulations on the waveforms *h*_1_(*n*), *h*_2_(*n*), *h*_3_(*n*), with *N* = 10^7^ samples, generated using the described simulation method. The corresponding SNRs are obtained using Equations (2) and (3) for the given average SNR and system parameters, the relay operated based on Equation (1), and the time series for the instantaneous capacity for the system, with the relaying and referent system being determined using Equations (15) and (16), respectively. The ergodic capacity is estimated via averaging the instantaneous capacity, i.e., the mean value on the corresponding waveform is determined.

The propagation conditions at both satellite–terrestrial links are varied, while the propagation conditions at the R-D link are fixed (*m*_3_ = 5 and Ω_3_ = 1). The threshold at the relay was set to γ*_th_*_,*R*_ = 0dB. The results for the system with relaying are compared to the numerical results obtained for the referent system, where only the S-D link is present. The theoretical results for the referent system can also be calculated using Equation (45) if we set γ*_th_*_,*R*_ = ∞, while, in our experiments, we set γ*_th_*_,*R*_ = 10,000.

In the case when heavy shadowing is present both at the S-D link and S-R link, an increase in the ergodic capacity due to relaying is visible for lower values of the average SNR. However, for high values of the average SNR, the effect of relaying disappears, and the referent system is superior for $\bar{\gamma }=20\;\mathrm{dB}$.

As expected, an additional gain is obtained if the heavy shadowing is present at the S-D link and the average shadowing is present at the S-R link. In such a case, the system with relaying provides a larger ergodic capacity for the whole practically important range of the average SNR.

Finally, we presented the numerical results for the scenario where the average shadowing is present both at the S-D link and the S-R link. In such a case, the referent system (S-D link only) for any value of the average SNR provides a larger ergodic capacity when compared to the system with relaying. In this case, the contribution of the MRC combiner is not enough to compensate for the inefficient usage of the bandwidth.

**Figure 14:**
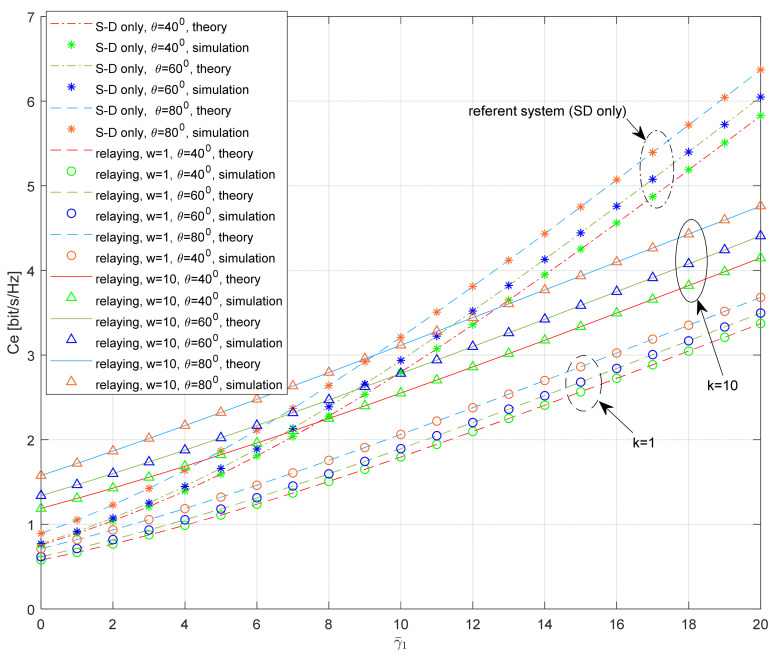
Ergodic capacity vs. average SNR; the impact of the system geometry.

The dependence of the average SNR on the ergodic capacity for various system geometry parameters is presented in Figure 14 for the average shadowing both in the S-D and S-R links. The analysis was performed based on the varying elevation angles of the satellite and the various distances between the relay and the destination. Also, the scenarios with various values of the scaling factor $w={\bar{\gamma }}_{3}/{\bar{\gamma }}_{1}$ are considered.

Obviously, the elevation angle has an impact on the distances between the satellite and the terrestrial user as *d_SD_* ≈ *d_SR_* = *H*/sin(θ). Furthermore, it is well known that the parameters of the shadowed Rice model can be determined as a function of the elevation angle of the satellite, based on the following expressions:

(66) $$\begin{array}{l} b_{0}(\theta )=-4.7943\times 10^{-8}\theta^{3}+5.5784\times 10^{-6}\theta^{2}-2.1344\times 10^{-4}\theta +3.2710\times 10^{-2},\\ m(\theta )=6.3739\times 10^{-5}\theta^{3}+5.8533\times 10^{-4}\theta^{2}-1.5973\times 10^{-1}\theta +3.5156,\\ \Omega (\theta )=1.4428\times 10^{-5}\theta^{3}-2.3798\times 10^{-3}\theta^{2}+1.2702\times 10^{-1}\theta -1.4864, \end{array}$$

and the above expressions are accurate for the elevation angles 20° ≤ θ ≤ 80° [12].

As expected, the numerical results indicate that the ergodic capacity of the system is reduced for smaller values of the elevation angle. This effect is visible for the system with relaying, as well as for the referent system. If ${\bar{\gamma }}_{1}=5\;\mathrm{dB}$, the ergodic capacity of the referent system (with S-D link only) is 1.6 bit/s/Hz for θ = 40°, and 1.85 bit/s/Hz for θ = 80°.

If the relaying is applied, and the average SNR is the same at the S-D and R-D link (i.e., *w* = 1), the ergodic capacity is smaller when compared to the referent system (due to the inefficient usage of the bandwidth). If ${\bar{\gamma }}_{1}={\bar{\gamma }}_{3}=5\;\mathrm{dB}$, the ergodic capacity of the system with relaying is equal to 1.34 bit/s/Hz for θ = 80°. If the average SNR in the terrestrial link is higher than the average SNR in the S-D link (i.e., *w* > 1), the ergodic capacity of the system can be further increased. The positive impact of the strong terrestrial link is especially visible for the low SNR values, and the impact of the elevation angle is more noticeable in the whole observed range of the average SNRs. If ${\bar{\gamma }}_{1}=5\;\mathrm{dB}$ and ${\bar{\gamma }}_{3}=10{\bar{\gamma }}_{1}$, the ergodic capacity of the system with relaying is equal to 2.33 bit/s/Hz for θ = 80°.

## 6. Conclusions

In this paper, a hybrid satellite–terrestrial relay network is analyzed, based on the DF selection relaying scenario. The system performances are analyzed for the case when the propagation in the satellite–terrestrial links can be described using the shadowed Rice statistical model, and when the terrestrial links undergo Nakagami-*m* fading. The novel closed-form expressions are derived for the outage probability, the outage capacity, and the ergodic capacity. The analytical expressions are derived in the polynomial–exponential form and verified using an independent Monte Carlo simulation method.

Based on the obtained results, in the case of small threshold values, the influence of the relaying is more visible for the light shadowing in satellite–terrestrial links. However, for larger threshold values, the system with relaying is inferior when compared to the referent system due to the inefficient bandwidth usage. Furthermore, the relaying increases the outage capacity for smaller values of the outage probability, and, for heavy shadowing, the positive impact of the relaying is more visible in the wide range of *P_out_*. In general, it can be noticed that the system performances are more sensitive to the quality of the terrestrial link if the propagation in the S-D and S-R links corresponds to the light shadowing scenario. In the case when the S-R link experiences better propagation conditions, the relaying is more effective for all analyzed values of the average SNR. On the other hand, the relaying increases the ergodic capacity of the system only for the heavy propagation at the satellite–terrestrial links and smaller values of the average SNR, and the effect of relaying is more visible if the average SNR in the terrestrial link can be additionally increased.

The provided analysis of the considered hybrid satellite–terrestrial relay network provides important guidelines for the design of future satellite–terrestrial networks, in which the communication links between the satellite and mobile terrestrial users can be unstable due to the multipath and shadowing effects. In our future work, the analysis will be extended to obtain the expressions for the bit error rate for the chosen modulation formats, and we will also investigate the physical layer security of the proposed system. Using the developed simulator, we will further extend the analysis presented in [59], where we analyzed a user-centric handover procedure, which can be used to improve the downstream communication from the LEO satellite network to end users. The proposed simulator will be used to determine the performances of the error-correction codes in the time-varying satellite–terrestrial channel, and it can be further modified to generate the waveforms that correspond to the alternative statistical models for the satellite–terrestrial links (e.g., gamma-gamma fading).

## Figures and Tables

**Table 1:** Table of symbols.

Symbol	Explanation
*P_S_*	the power of the signal from the satellite
*P_R_*	the power the signal from the relay
*h*_1_	the complex channel gain between the satellite (S) and destination (D)
*h*_2_	the complex channel gain between the satellite (S) and relay (R)
*h*_3_	the complex channel gain between the relay (R) and destination (D)
γ_1_	the instantaneous SNR at the output of the S-D link
γ_2_	the instantaneous SNR at the output of the S-R link
γ_3_	the instantaneous SNR at the output of the R-D link
γ	the instantaneous SNR at the output of the MRC combiner at the destination
γ_th_	the threshold at the destination
γ_th,R_	the threshold at the relay
*d*_SD_	the distance between the satellite and destination
*d*_SR_	the distance between the satellite and relay
*d*_RD_	the distance between the relay and destination
*n*_SD_	the path loss between the satellite and destination
*n*_SR_	the path loss between the satellite and relay
*n*_RD_	the path loss between the relay and destination
θ	elevation
σ^2^	the noise power at the destination
$\lambda_{i}$	the normalized power gain in the *i*-th channel
${\bar{\gamma }}_{i}$	the average signal–noise ratio at the output of the *i*-th channel
*m_i_*	the Nakagami fading parameter at the *i*-th channel
Ω*_i_*	the average power of the LOS component in the *i*-th channel
*b*_0,*i*_	the average power of the scattering component in the satellite–terrestrial links
*f_Dm_*	the maximum Doppler shift for the multipath in the S-D and S-R links
*f_Ds_*	the maximum Doppler shift for the shadowing in the S-D and S-R links
*f_Dt_*	the maximum Doppler shift for the terrestrial link
*f*_0_	the carrier frequency at the satellite–terrestrial links
*c*	the speed light
*C*	the instantaneous capacity
*C_out_*	the outage capacity
*C_e_*	the ergodic capacity
*C_th_*	the capacity threshold
*P_out_*	the outage probability
*EIRP*	the effective isotropic radiated power
*L_A_*	denotes the typical loss due to the atmospheric conditions
*G*	the antenna gain at the receiver
*T_S_*	the temperature of the system
*B*	the channel bandwidth
*k*	the Boltzmann constant

**Table 2:** System and simulation parameters.

System Parameters	*Value*	Simulation Parameters	*Value*
*EIRP*	36.7 dBW	*P_S_*	9.9 dBW
*L_A_*	14 dB	σ^2^	−89 dBm
*G*	10.5 dB	*n_SD_*, *n_SR_*	2
*T_s_*	363 K	*H*	550 km
*B*	250 MHz	θ	60°
*f*_0_	11 GHz	*L*	25 km

**Table 3:** Typical channel parameters.

Propagation Scenario	*b*_0_	*m*	Ω
Infrequent light shadowing	0.158	19	1.29
Average shadowing	0.126	10	0.835
Frequent heavy shadowing	0.063	1	0.000897

## Data Availability

Data is contained within the article.

## Appendix A

The inner integral in (27) corresponds to the cumulative density function of the instantaneous SNR at the terrestrial link, which can be represented in the following form [28]:

(A1) $$\int_{0}^{\gamma_{th}-\gamma_{1}}f_{\Gamma_{3}}(\gamma_{3})d\gamma_{3}=1-\frac{\Gamma \left(m_{3},\frac{m_{3}}{\Omega_{3}{\bar{\gamma }}_{3}}(\gamma_{th}-\gamma_{1})\right)}{\Gamma \left(m_{3}\right)}$$

Therefore, the integral *I*_4_ can be split into two terms as follows:

(A2) $$I_{4}=\int_{0}^{\gamma_{th}}f_{\Gamma_{1}}(\gamma_{1})\;d\gamma_{1}-\int_{0}^{\gamma_{th}}f_{\Gamma_{1}}(\gamma_{1})\frac{\Gamma \left(m_{3},\frac{m_{3}}{\Omega_{3}{\bar{\gamma }}_{3}}(\gamma_{th}-\gamma_{1})\right)}{\Gamma \left(m_{3}\right)}\;d\gamma_{1}.$$

The numerical solution of the above integrals, for the arbitrary values of the fading parameters in the S-D and R-D links and the special case when ${\bar{\gamma }}_{3}=1$, was considered in the paper [33].

The first integral on the right-hand side in (A2), for the special case, when *m*_1_ is integer valued, was already derived in (24) in the following closed form:

(A3) $$\int_{0}^{\gamma_{th}}f_{\Gamma_{1}}(\gamma_{1})\;d\gamma_{1}=1-\alpha_{1}e^{-\frac{\beta_{1}-\delta_{1}}{{\bar{\gamma }}_{1}}\gamma_{th}}\sum_{k_{1}=0}^{m_{1}-1}\frac{\varsigma_{1}(k_{1})}{{\bar{\gamma }}_{1}^{k_{1}+1}}\sum_{p_{1}=0}^{k_{1}}\frac{k_{1}!}{p_{1}!}{\left(\frac{\beta_{1}-\delta_{1}}{{\bar{\gamma }}_{1}}\right)}^{-(k_{1}+1-p_{1})}\gamma_{th}^{p_{1}}.$$

On the other hand, the second integral on the right-hand side in (A2) can be further simplified for the integer-valued parameter *m*_3_ through the use of expression (22) and the fact that $\Gamma (m_{3})=(m_{3}-1)!$ [46]; from this, we obtain

(A4) $$\int_{0}^{\gamma_{th}}f_{\Gamma_{1}}(\gamma_{1})\frac{\Gamma \left(m_{3},\frac{m_{3}}{\Omega_{3}{\bar{\gamma }}_{3}}(\gamma_{th}-\gamma_{1})\right)}{\Gamma \left(m_{3}\right)}\;d\gamma_{1}=\alpha_{1}e^{-C\gamma_{th}}\sum_{k=0}^{m_{1}-1}A_{k}\sum_{p=0}^{m_{3}-1}\frac{C^{p}}{p!}\int_{0}^{\gamma_{th}}\gamma_{1}^{k}e^{-(B-C)\gamma_{1}}{\left(\gamma_{th}-\gamma_{1}\right)}^{p}d\gamma_{1},$$

where

(A5) $$A_{k}=\frac{\varsigma_{1}(k)}{{\bar{\gamma }}_{1}^{k+1}},\;B=\frac{\beta_{1}-\delta_{1}}{{\bar{\gamma }}_{1}},\;C=\frac{m_{3}}{\Omega_{3}{\bar{\gamma }}_{3}}.$$

Finally, after applying the binomial formula, the integral in the right-hand side of (A4) can be solved as follows [60]:

(A6) $$\begin{array}{c} \int_{0}^{\gamma_{th}}\gamma_{1}^{k}e^{-(B-C)\gamma_{1}}{\left(\gamma_{th}-\gamma_{1}\right)}^{p}d\gamma_{1}=\sum_{q=0}^{p}\left(\begin{array}{c} p\\ q \end{array}\right)\gamma_{th}^{p-q}{\left(-1\right)}^{q}\int_{0}^{\gamma_{th}}\gamma_{1}^{k+q}e^{-(B-C)\gamma_{1}}d\gamma_{1}\\ \;\;=\sum_{q=0}^{p}\left(\begin{array}{c} p\\ q \end{array}\right)\gamma_{th}^{p-q}{\left(-1\right)}^{q}\times \left[\frac{(k+q)!}{{\left(B-C\right)}^{k+q+1}}-e^{-(B-C)\gamma_{th}}\sum_{j=0}^{k+q}\frac{(k+q)!}{j!}\frac{\gamma_{th}^{j}}{{\left(B-C\right)}^{k+q-j+1}}\right]. \end{array}$$
